# All metrics are equal, but some metrics are more equal than others: A systematic search and review on the use of the term ‘metric’

**DOI:** 10.1371/journal.pone.0193861

**Published:** 2018-03-06

**Authors:** Núria Duran Adroher, Birgit Prodinger, Carolina Saskia Fellinghauer, Alan Tennant

**Affiliations:** 1 Swiss Paraplegic Research, Nottwil, Switzerland; 2 Department of Health Sciences and Health Policy, University of Lucerne, Lucerne, Switzerland; 3 Faculty of Applied Health and Social Sciences, University of Applied Sciences Rosenheim, Rosenheim, Germany; Wenzhou Medical University Eye Hospital, CHINA

## Abstract

**Objective:**

To examine the use of the term ‘metric’ in health and social sciences’ literature, focusing on the interval scale implication of the term in Modern Test Theory (MTT).

**Materials and methods:**

A systematic search and review on MTT studies including ‘metric’ or ‘interval scale’ was performed in the health and social sciences literature. The search was restricted to 2001–2005 and 2011–2015. A Text Mining algorithm was employed to operationalize the eligibility criteria and to explore the uses of ‘metric’. The paradigm of each included article (Rasch Measurement Theory (RMT), Item Response Theory (IRT) or both), as well as its type (Theoretical, Methodological, Teaching, Application, Miscellaneous) were determined. An inductive thematic analysis on the first three types was performed.

**Results:**

70.6% of the 1337 included articles were allocated to RMT, and 68.4% were application papers. Among the number of uses of ‘metric’, it was predominantly a synonym of ‘scale’; as adjective, it referred to measurement or quantification. Three incompatible themes ‘only RMT/all MTT/no MTT models can provide interval measures’ were identified, but ‘interval scale’ was considerably more mentioned in RMT than in IRT.

**Conclusion:**

‘Metric’ is used in many different ways, and there is no consensus on which MTT metric has interval scale properties. Nevertheless, when using the term ‘metric’, the authors should specify the level of the metric being used (ordinal, ordered, interval, ratio), and justify why according to them the metric is at that level.

## Introduction

The term ‘metric’ is widely used in various fields of science, and it has gained different meanings with the years. These meanings have given rise to different perceptions on what is implied by its subsequent use. Consequently, it is important to understand how the term ‘metric’ is being used, and what characteristics are associated with these different meanings.

In the 19th century, when it was first used, it referred mainly to the metric system [1]. In the first half of the 20th century one can find also articles using it in the mathematical sense of metric (or distance) function (see S1 Appendix and [2]).

The term ‘scale’ also appears in the 19th century’s literature—e.g., Stiff [1] used it as a way to calculate weights and measures. In the mid-20th century, Coombs [3] brought together ‘metric’ and ‘scale’ introducing the term ‘ordered metric’ as a new type of scale to the four distinguished by Stevens [4]: *It is interesting to note here that this is a new type of scale not discussed by Stevens*. *This is a type of scale that falls between what he calls ordinal scales and interval scales*. *In ordinal scales nothing is known about the intervals*. *In interval scales the intervals are equal*. *In this scale*, *which we call an ordered metric*, *the intervals are not equal but they may be ordered in magnitude*. In other words, in an ordered metric scale one is able to order the distances between some pairs of points. For instance, one may say that the distance between two points A and B is bigger than the distance between two other points C and D. Therefore, one has more information than in an ordinal scale but less than in an interval scale.

In the second half of the 20th century, a distinction between ‘nonmetric’ and ‘metric’ was made. The former refers to ordinal data and the later to interval or ratio data, as Takane et al. [5] explained: *Previous authors of multidimensional scaling papers have emphasized a dichotomy of measurement levels which they termed metric and nonmetric*. *When placed in the context of Stevens* [6] *measurement theory*, *it is clear that these terms correspond to three of the four measurement levels delineated by Stevens*, *namely ordinal (nonmetric) and interval or ratio (metric)*.

Also in the second half of the last century, ‘metric’ has been used in the sense of statistic or index. Schuman and Brace [7] assessed the metric variations of chimpanzee dentition, where ‘metric’ *consists of recording of dimensions*: *their means*, *ranges*, *standard deviations*, *coefficients of variation*, *modules*, *indices* (*talonid-trigonid*, *length-breadth*).

More recently, these different uses of the term ‘metric’ are found in the health and social sciences. For example, Crane et al. [8] use ‘metric’ practically as a synonym of ‘scale’. Moreover, Kemmler et al. [9] point out the distinction between metric scaling and ordinal scaling, and Arons and Krabbe [10] investigated both metric and nonmetric multidimensional scaling. In the sense of statistic or index, Bai et al. [11] present the Activity Index as a new metric for summarizing raw tri-axial accelerometry data.

Modern Test Theory (MTT) models—consisting of the Rasch Measurement Theory (RMT) [12] and Item Response Theory (IRT) [13] paradigms [14], are used in the health and social science’s literature, inter alia, to evaluate Patient Reported Outcome Measures (PROMs) [15]. The term ‘metric’ is widely used in MTT, however, it is rarely defined. Based on this historical overview on the use of the term ‘metric’, it seems that its use predominantly implies interval scaling. For researchers, this would imply that they can use parametric methods for analyzing the respective data given appropriate distributions. However, if the term is vaguely defined and does not have interval scale properties but researchers treat it as such, there is a risk of getting erroneous results [16] or of under- or overestimating effects in e.g. clinical research [17]. Clinicians dealing with indexes such as Minimal Clinical Important Difference (MCID) in PROMs should be aware that ordinal-based derivations of MCID can lead to wrong conclusions [17]. Kahler et al. [16] provided an example showing the inappropriateness of applying parametric methods to Health-Related Quality of Life scores, i.e., to ordinal data. While it is clear that when using the term ‘interval scale’ mathematical calculations such as the mean and standard deviation are allowed, it is not so clear if the term used is ‘metric’. Therefore, if a researcher or clinician encounters the term ‘metric’, they do not know if they are allowed to perform arithmetic operations. It is also not so clear if MTT models can transform PROM scores to interval data. The current study aims to put some light on these issues.

### Objective

The objective of this paper is to examine the use of the term ‘metric’ in health and social sciences’ literature, focusing on the interval scale implication of the term in MTT. Specifically, we aim (1) to explore the different uses and meanings of ‘metric’ in MTT literature, (2) to examine the relationships between ‘metric’ and ‘interval scale’ in RMT and IRT, and (3) to compare the current understanding on whether either or both paradigms can produce interval scaling.

## Materials and methods

A Systematic Literature Search and Review [18] was carried out, and the search, data extraction, and data synthesis processes are explained as follows. Where appropriate, this review follows the PRISMA guidelines (see [19] and S2 Appendix). The detailed review protocol is available at S3 Appendix.

### Systematic Literature Search

**Fig 1:**
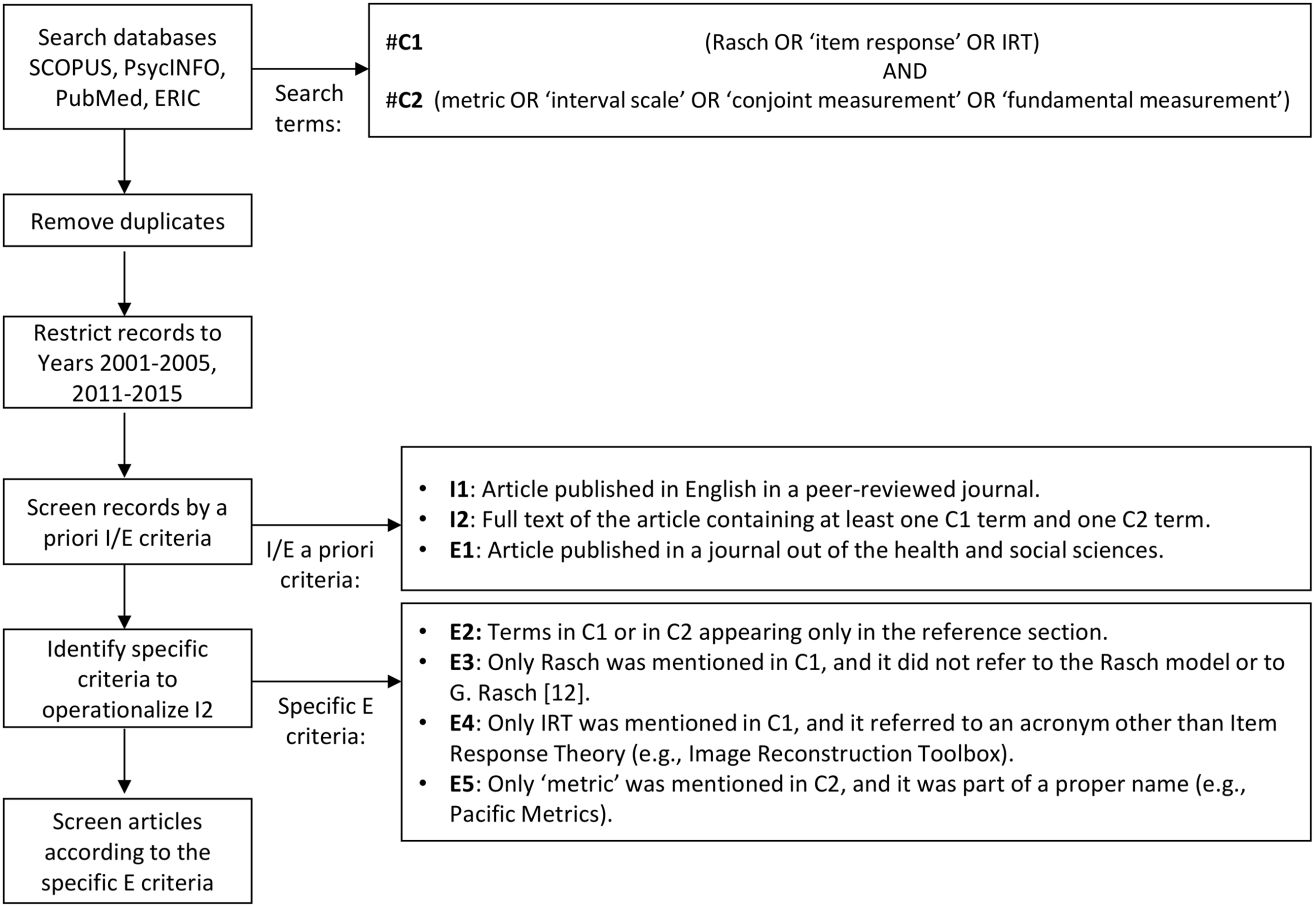
Iterative process of the Systematic Literature Search. *Abbreviations*: IRT, Item Response Theory; C, Combination; I, Inclusion; E, Exclusion.

Fig 1 illustrates the iterative process of the Systematic Literature Search. The databases SCOPUS, PsycINFO, PubMed and ERIC were systematically searched. The search terms used in combination in all the databases are shown at the top of Fig 1. Combination 1 (C1) enabled to restrict the articles to MTT literature. Combination 2 (C2), apart from ‘metric’ and ‘interval scale’, whose relationship aimed to be examined, also included ‘conjoint measurement’ and ‘fundamental measurement’. The theory of Conjoint Measurement (CM) provides a means to quantify attributes, i.e., to obtain interval measures [20]. Therefore, CM can be used to justify interval measurement. In fact, the Rasch Measurement Model has been argued to be a probabilistic form of Additive CM [21]. As Simultaneous CM is a form of Fundamental Measurement [20], the latter was also included.

**Fig 2:**
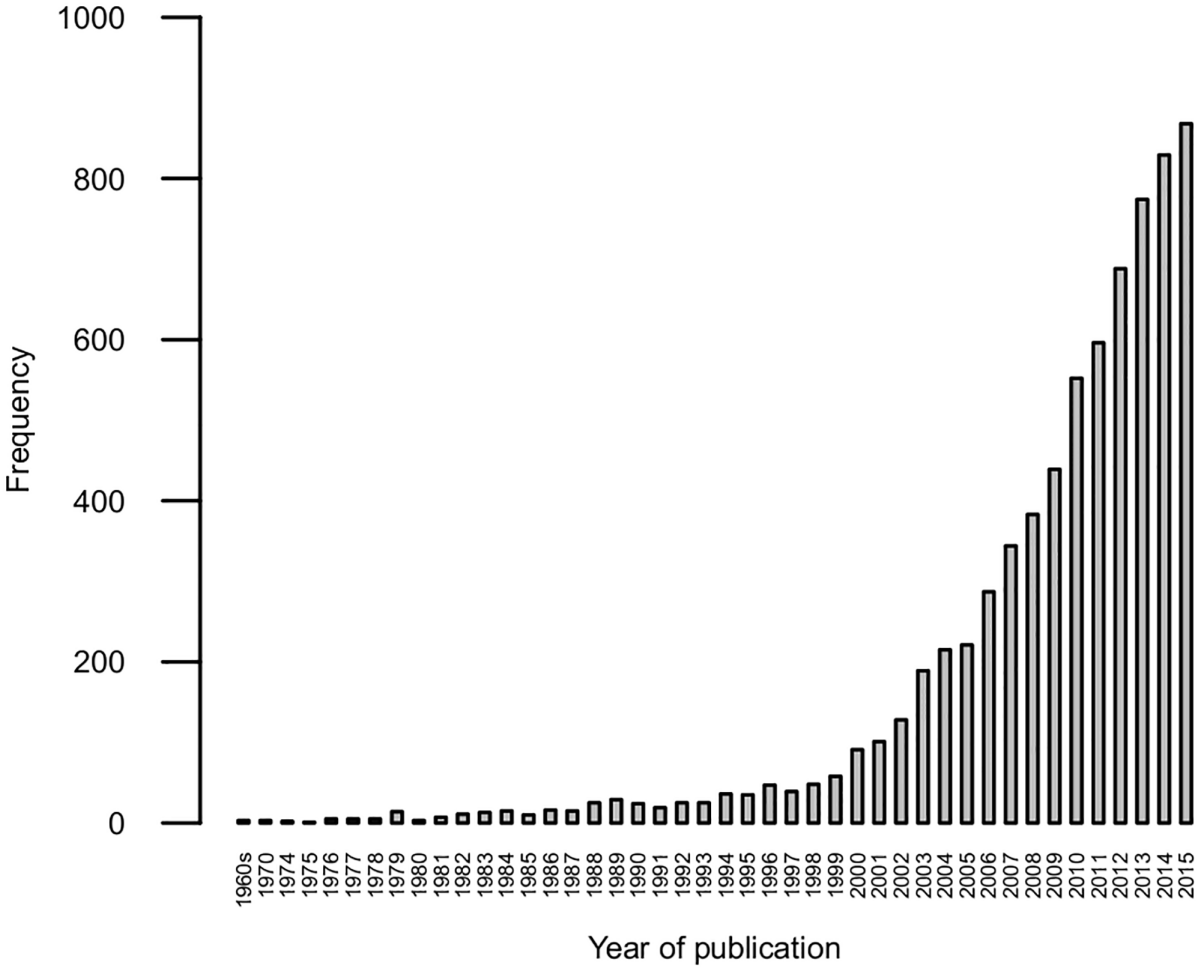
Barplot of the 7243 records identified through database search per year.

A year restriction was established due to the vast amount of records identified in the database search. As there was a clear increase in the number of records after 2001 as shown in Fig 2, we decided to consider the articles published from 2001 to 2005 and from 2011 to 2015. We considered two periods (2001–2005 and 2011–2015) hypothesizing that relevant findings from papers published previous to 2001 would be cited in the 2001–2005 period, and that relevant findings from papers published in 2006–2010 would be cited in 2011–2015.

The records were subsequently screened according to the a priori inclusion and exclusion criteria shown in Fig 1. Inclusion criterion 1 (I1) and Exclusion criterion 1 (E1) were checked manually, and I2 by means of a Text Mining (TM) strategy using R software v.3.3.0 (see S4 Appendix). The TM strategy was applied and refined in a few iterations before it was applied to all the eligible articles. In this iterative process, four additional specific Exclusion criteria (E2–E5) were identified to operationalize I2 (see Fig 1).

As regards E2, if terms in C1 (e.g., Rasch) or in C2 (e.g., fundamental measurement) only appeared in the reference section, they were not used in the main manuscript texts, letting us to conclude that the paper did not deal substantially with the topic under review in our study. If E3–E5 were applicable, it followed that I2 could not be satisfied any more and therefore the article was neither included.

The TM algorithm described in S4 Appendix includes a quality check of the fulfillment of the eligibility criteria.

### Data extraction and data synthesis

To respond to Aim 1, there was no need to classify the included articles, because for all of them, the previous and posterior context of the uses of ‘metric’ were examined. This information was obtained applying the TM strategy. Once this information was processed, the uses of ‘metric’ were distinguished as parts of the speech: noun, adjective or adverb. Synonyms and definitions of ‘metric’ were also collected.

To respond to Aim 2, the RMT and IRT paradigms needed to be distinguished to examine the uses of ‘metric’ and ‘interval scale’ in each of the paradigms. If in the article only RMT was mentioned, the paradigm was clearly RMT, and if only IRT was mentioned, the paradigm was IRT. If both terms were mentioned, then the paradigm could be RMT, IRT, or MTT, depending on what the article was dealing with. If the article was mainly dealing with RMT, then RMT was the paradigm, and the same holds true for IRT. If both had a similar presence, then the article was allocated to a global group, MTT. Each article was allocated to one paradigm, and this information was added to a table indicating if each article contained the terms ‘metric’ and ‘interval scale’. Contingency tables on the individual and combined usages of ‘metric’ and ‘interval scale’ by paradigm could then be calculated.

To respond to Aim 3, in order to identify the reasons why either or both RMT and IRT models can produce interval scaling, a more in depth review of the included articles was needed. Due to the high amount of included articles, we selected a sample of them and we examined how they could be categorized into meaningful groups. Most of them reported on studies in which RMT or IRT models were applied to validate, co-calibrate or develop an instrument, which we referred to *Application* papers. There were also *Theoretical* papers, dealing with abstract measurement discussions, and not involving in general any data analysis. Moreover, there were some which were neither pure application nor theory, but examined different RMT or IRT *Methodologies* via simulation, or presented a novel method, containing some theory but also an application to test the methodology. Another type referred to articles which did not present an application, a methodology nor discussed in depth theoretical measurement issues; but they explained aspects of RMT or IRT with an educational (*Teaching*) aim. Finally, a last group consisted of articles which mentioned RMT or IRT, predominantly only in the introduction or discussion sections, and their main focus was not on MTT, belonging therefore to a *Miscellaneous* category. After having identified these five article typologies (*Theoretical*, *Methodological*, *Teaching*, *Application*, and *Miscellaneous*) based on a sample of the included articles, we hypothesized that the whole set of the articles could be assigned to one of the previous five types, and the article type allocation was also performed.

Two of the authors, NDA and CSF, performed manually both the paradigm and type allocation simultaneously based on the abstract. In case of ambiguity, the full texts were consulted. The two authors first performed both allocations in a random sample of 100 articles and then the disagreements were discussed. Then the remaining articles were split amongst NDA and CSF who performed the allocations of the remaining articles separately. The detailed guidelines for paradigm and type allocation are listed in S5 Appendix.

While allocating each article to one paradigm and one type, some comments were made if the article could potentially contribute to justify the ability of RMT and IRT to obtain interval measures. The highlighted articles virtually belonged to *Theoretical*, *Methodological*, and *Teaching* types. Therefore, it was agreed by the research team that *Application* and *Miscellaneous* articles would not provide in-depth information in response to Aim 3; thus, it was decided that only *Theoretical*, *Methodological*, and *Teaching* articles would be included in the further full text review. NDA extracted text passages which referred to the ability of RMT and IRT to obtain interval measures into a Word document. Then NDA conducted an inductive thematic analysis [22] in the extracted texts identifying different themes which were then discussed with the research team and subsequently refined.

## Results

**Fig 3:**
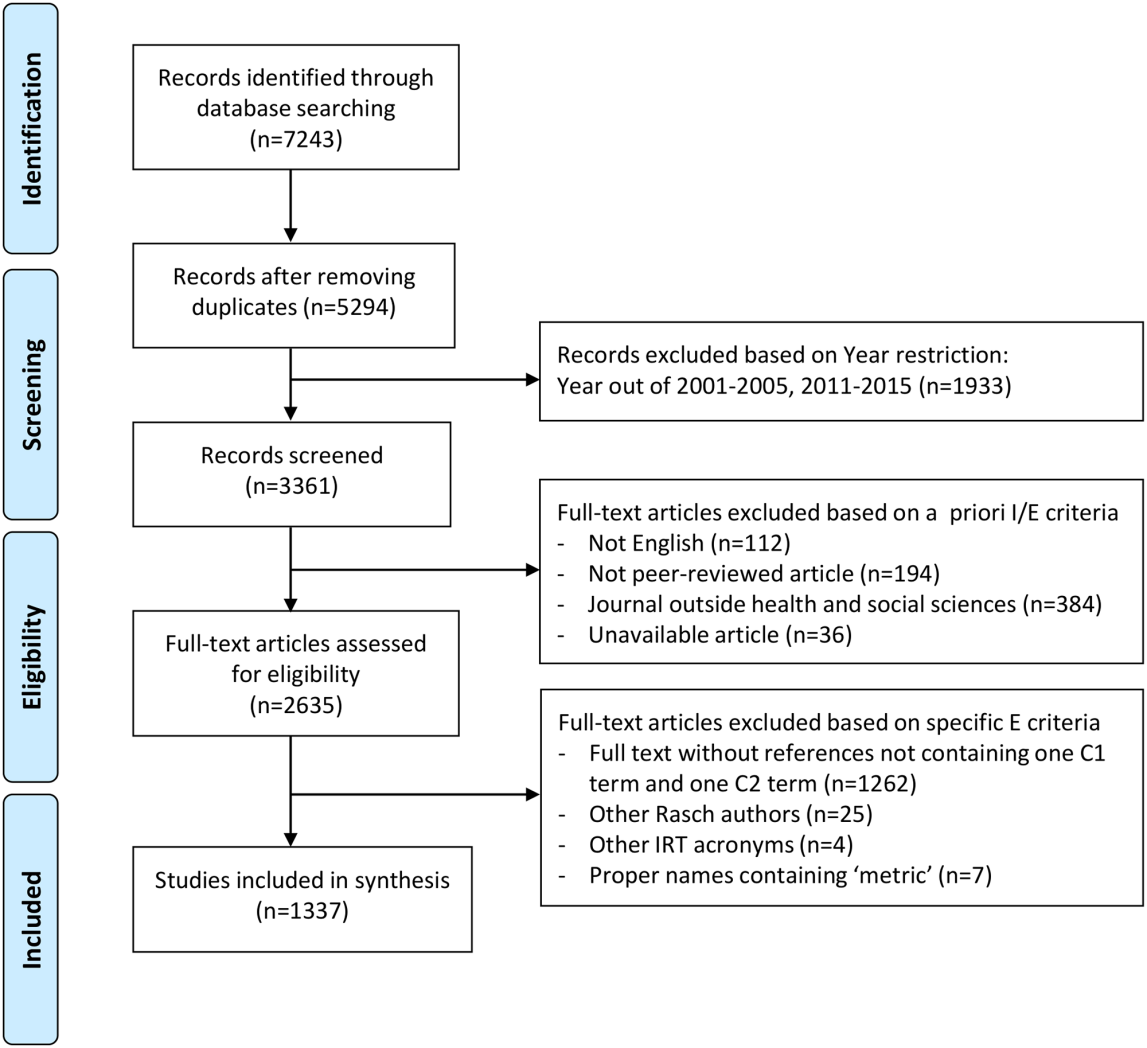
Flow diagram of the search and eligibility processes. *Abbreviations*: C, Combination; IRT, Item Response Theory.

Initially, 7243 records were identified (4594 in Scopus, 2135 in PsycINFO, 294 in ERIC, and 220 in PubMed). From these, 1949 were duplicates. After applying the year restriction, we ended up with 3361 records to screen. Out of those, 690 were excluded because they were not written in English, were not peer-reviewed articles, or belonged to journals outside the health and social sciences. There were 36 articles which were unavailable to us. The exact figures are shown in Fig 3.

The 2635 eligible articles were assessed for eligibility by means of the Text Mining strategy detailed in S4 Appendix. This step in the literature search resulted in 1337 articles. The dataset containing the included articles is available at S6 Appendix.

The agreement in the pilot sample of 100 articles between the two authors for paradigm allocation was 92%, and for type allocation 79%. Conflicts in type allocation concerned mainly the *Miscellaneous* type. The criteria for type allocation were refined accordingly. Only then the remaining articles were split amongst the two authors who performed paradigm and type allocations separately.

**Fig 4:**
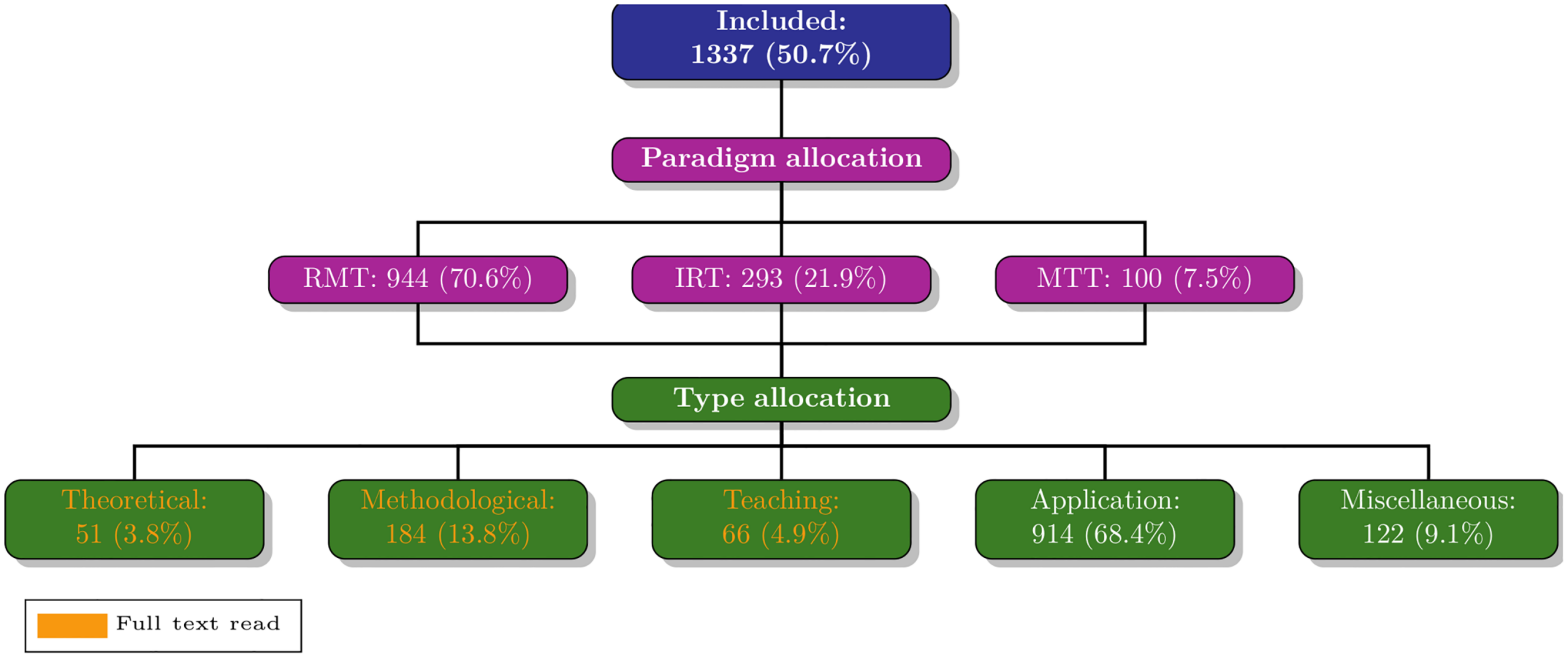
Flow chart of the data extraction process. *Abbreviations*: RMT, Rasch Measurement Theory; IRT, Item Response Theory; MTT, Modern Test Theory.

The majority (70.6%) of the included articles were allocated to the RMT paradigm and 21.9% to the IRT paradigm (see Fig 4). The Type allocation resulted in the clear predominance of *Application* studies (68.4%), followed by the types *Methodological* (13.8%), *Miscellaneous* (9.1%), *Teaching* (4.9%), and finally *Theoretical* studies (3.8%).

As it can be seen in Table 1, most of the RMT articles were Application studies (84.2%), while for IRT one third were *Application* and one third *Methodological* studies. A higher proportion for *Miscellaneous* articles was found for IRT than for RMT.

**Table 1:** Contingency table of paradigm by Type.

Paradigm	Theoretical	Methodological	Teaching	Application	Miscellaneous	Total
RMT	22 (2.3%)	59 (6.2%)	26 (2.8%)	795 (84.2%)	42 (4.4%)	944
IRT	7 (2.4%)	95 (32.4%)	18 (6.1%)	103 (35.2%)	70 (23.9%)	293
MTT	22 (22%)	30 (30%)	22 (22%)	16 (16%)	10 (10%)	100
Total	51 (3.8%)	184 (13.8%)	66 (4.9%)	914 (68.4%)	122 (9.1%)	1337

*Abbreviations*: RMT, Rasch Measurement Theory; IRT, Item Response Theory; MTT, Modern Test Theory.

### Uses and meanings of ‘metric’

Table 2 shows the previous and posterior context of some of the uses of ‘metric’ as a noun and as an adjective or adverb, containing an example of a reference where it was used. Table 3 contains meanings (synonyms and definitions) of ‘metric’, also with the corresponding reference. The complete list of uses, synonyms and definitions can be found in S7 Appendix.

**Table 2:** Examples of uses of ‘metric’. First as a noun, then as adjective/adverb.

Pre	Term	Post	Reference
ability	metric		[29]
latent	metric		[30]
observed score	metric		[31]
raw/raw score/raw-score interval	metric		[32]/ [33]/ [34]
difficulty	metric		[35]
	metric	of the items	[36]
Euclidian	metric		[37]
IRT	metric		[38]
logistic/logit/log-odds	metric		[39]/ [40]/ [41]
PROMIS	metric		[42]
Rasch	metric		[43]
T/T-score	metric		[44]/ [45]
z/z-score	metric		[46]/ [47]
Equating the two	metrics	placed the item parameter estimates from both samples on the same scale.	[48]
common	metric		[49]
better/good/more appropriate/strongest possible	metric		[50]/ [51]/ [52]/ [53]
ordinal	metric		[54]
quasi-interval	metric		[55]
interval	metric		[56]
linear	metric		[57]
quantitative	metric		[58]
cardinal	metric		[59]
continuous	metric		[60]
absolute	metric		[60]
transform raw ordinal scores into an interval	metric		[61]
[…] can depend on the	metric	used for the analysis (e.g., scale scores, standardized scores).	[23]
arbitrary	metric		[24]
unit of a	metric		[62]
ordered	metric	scale	[63]
[…] are required to obtain ordered-	metric	scales for respondents and items that place in-between ordinal and interval metrics.	[25]
quasi-	metric	scale	[64]
	metric	scale	[65]
	metric	continuum	[66]
	metric	score	[67]
	metric	variables	[26]
Types of variables used in scales: ordinal, nominal,	metric		[68]
	metric	level	[69]
or somehow forcing them [the measurements] to be expressed	metrically	by employing, for example, additive conjoint measurement.	[26]
	metric	properties	[70]
well-defined labor quality attributes with	metric	properties (i.e., with well-defined unit of measure and zero).	[71]
non	metric	properties of ordered categorical data	[27]
[…] the order of all item difficulties and individual abilities remains the same across all locations of the scale *θ*. This preservation of	metric	properties across the scale is of utmost importance for the scale anchoring procedures used in this study.	[28]
better/good/ideal/optimal/poor/superior	metric	properties	[72]/ [73]/ [74]/ [75]/ [76]/ [77]
	metric	quality	[78]
	metric	ruler	[79]
	metric	distortion	[80]

**Table 3:** Synonyms and definitions of ‘metric’.

Pre	Term	Post	Reference
continuum or	metric		[84]
continuous (	metric	)	[85]
	metric	or interval scale	[17]^1^
measure or	metric		[86]
	metric	or scale	[87]
ratio scaled (	metric	)	[26]
	Metric	: The underlying value that is used to understand the score and how it is scaled so that meaning can be derived from a score. For the SCI-QOL, the reported metric is a ‘T Metric’ with an average of 50 and standard deviation unit of 10. For PROMIS, the metric reflects the general population average. For SCI-QOL, the metric reflects either general population (when anchored to a PROMIS or Neuro-QOL scale) or to the SCI-population (when it is a new bank that does not have a comparable PROMIS or Neuro-QOL bank). The metric is relative the population that was used to calibrate the items.	[81]
	metrics	(i.e., scales for continuous quantities in well-defined units).	[82]
a scale can be: […] (3) a system of units and numbers that define a mathematical	metric	(e.g., feet and inches are units of the imperial scale; centimeters and meters are units of the metric scale).	[83]
place on a	metric	having a mean of zero and a standard deviation of one.	[88]

^1^Its publication date is 2016, but Epub 2015. This article and three more were identified as a 2015 article when the search was performed.

Eight groups of usages have been identified: 4 relating to ‘metric’ as a noun, 3 to ‘metric’ as an adjective/adverb, and 1 overlapping both.

#### ‘Metric’ as a noun

A first group of metric usages referred to the values that persons or items can take on the measurement continuum. Concerning persons, we found ‘ability metric’, ‘latent metric’, ‘observed score metric’, ‘raw metric’/‘raw score metric’/‘raw-score interval metric’; and concerning items, ‘difficulty metric’, ‘metric of the items’.

A second group indicated specific metrics, such as ‘Euclidian metric’, ‘IRT metric’, ‘logistic metric’, ‘logit metric’, ‘log-odds metric’, ‘PROMIS metric’, ‘Rasch metric’, ‘T metric’/‘T-score metric’, ‘z metric’/‘z-score metric’.

A third group was related to linking or equating metrics (‘Equating the two metrics placed the item parameter estimates from both samples on the same scale’), being ‘common metric’ the most prevalent term.

A fourth group contained adjectives qualifying ‘metric’ in a way that a gradient could be considered: ‘good/better/more appropriate/strongest possible metric’.

A fifth group consisted of another gradient, in this case describing different levels of a metric: ‘ordinal metric’, ‘quasi-interval metric’, ‘interval metric’, ‘linear metric’, ‘quantitative metric’, ‘cardinal metric’, ‘continuous metric’, ‘absolute metric’. There was a distinction between ordinal and interval levels in ‘Transform raw ordinal scores into an interval metric’: ordinal qualified ‘score’ and interval qualified ‘metric’. In contrast, Robinson and Lubienski [23] considered ‘scale scores’ and ‘standardized scores’ as two metrics; in addition, ‘raw metric’ also appeared in the first group of usages. Also, sometimes the Rasch model was presented as an application helping to overcome the ‘arbitrary metrics’ problem in the observed score metric used in Classical Test Theory [24].

#### ‘Metric’ as an adjective/adverb

The terms ‘ordered metric scale’, ‘quasi-metric scale’ and ‘metric scale’ could be part of the gradient in the fifth group. Noventa et al. [25] placed ‘ordered metric scale’ between ordinal and interval metrics. The last two terms would be equivalent to ‘quasi-interval metric’ and ‘interval metric’, respectively.

A sixth group of usages considered ‘metric’ as an indicator that the corresponding substantive was quantitative: ‘metric continuum’, ‘metric score’, ‘metric variables’. In this lines, ‘Types of variables used in scales: ordinal, nominal, metric’, ‘metric’ refers to interval and ratio variables. These variables are therefore at the ‘metric level’. Similarly, Krause [26] employs ‘metrically’ as a synonym of quantitatively: additive conjoint measurement is a method which enables to express measurements metrically.

A seventh group consisted of ‘metric properties’ uses. Sometimes it related to having quantitative properties (‘with a well-defined unit of measure and zero’, or the fact that ordered categorical data did not have metric properties [27]). Nevertheless, Hartig et al. [28] used ‘preservation of metric properties’ in the sense of preserving the order of item difficulties and individual abilities across the scale. A gradient for ‘metric properties’ was observed, too: ‘poor/good/better/superior/optimal/ideal metric properties’.

A final group of usages related to measurement itself: ‘metric quality’ seemed to refer to the quality of measurement; ‘metric ruler’ was used as a synonym of the item location map; and ‘metric distortion’ referred to the stretching or compression of the ‘units of a metric’.

#### Synonyms and definitions of ‘metric’

The following synonyms of ‘metric’ were observed: ‘continuum’, ‘continuous’ (adjective), ‘interval scale’, ‘measure’, ‘ratio scaled’ (adjective), ‘scale’.

Tulsky et al. [81] defined ‘metric’ explicitly as *the underlying value that is used to understand the score and how it was scaled so that meaning can be derived from a score*. Here it was also stated that *the metric is relative the population that was used to calibrate the items*. From his side, Kyngdon [82] defined ‘metrics’ as *scales for continuous quantities in well-defined units*. Moreover, one of the meanings of scale that Cook et al. [83] listed, one referred to *a system of units and numbers that defines a mathematical metric*.

Finally, a metric was sometimes determined as having a specific mean (e.g., 0) and standard deviation (e.g., 1).

### Relationship between ‘metric’ and ‘interval scale’

How the terms ‘metric’, ‘interval scale’, ‘conjoint measurement’, and ‘fundamental measurement’ were used within RMT and IRT is presented in Table 4. The descriptive statistics show that ‘interval scale’ was more often used in RMT than ‘metric’, 716 (76%) articles containing ‘interval scale’ vs 413 (44%) articles containing ‘metric’, in comparison to 37 (13%) vs 275 (94%) in IRT. ‘Conjoint measurement’ and ‘fundamental measurement’ were hardly used in the IRT paradigm, more commonly in the context of RMT or both RMT and IRT (MTT).

**Table 4:** Contingency table of search terms by paradigm.

Paradigm	Metric	Interval Scale	Conjoint Measurement	Fundamental Measurement	Total
RMT	413 (43.8%)	716 (75.8%)	78 (8.3%)	106 (11.2%)	944
IRT	275 (93.9%)	37 (12.6%)	4 (1.4%)	4 (1.4%)	293
MTT	63 (63%)	54 (54%)	27 (27%)	15 (15%)	100
Total	751 (56.2%)	807 (60.4%)	109 (8.2%)	125 (9.3%)	1337

*Abbreviations*: RMT, Rasch Measurement Theory; IRT, Item Response Theory; MTT, Modern Test Theory. The percentages are by row. Note that the sum by rows exceeds the respective Total, because an article can contain more than one term.

Out of the 751 articles that used ‘metric’, the use of ‘metric’ alone and in combination with ‘interval scale’ was balanced within the RMT paradigm and in those papers with a similar presence of both RMT and IRT (MTT). In contrast, there was a clear predominance of only using ‘metric’ rather than in combination with ‘interval scale’ in IRT (Table 5).

**Table 5:** Contingency table of the combined use of ‘metric’ and ‘interval scale’ by paradigm.

Paradigm	Metric	Metric with interval scale	Metric without interval scale
RMT	413	216 (52.3%)	197 (47.7%)
IRT	275	21 (7.6%)	254 (92.4%)
MTT	63	26 (41.3%)	37 (58.7%)
Total	751	263 (35%)	488 (65%)

*Abbreviations*: RMT, Rasch Measurement Theory; IRT, Item Response Theory; MTT, Modern Test Theory.

### Ability to produce interval scaling

In respond to the third specific aim, whether interval measures can be obtained with RMT or IRT models, three incompatible answers were derived from the thematic analysis: (1) only RMT models can provide interval measures, (2) both RMT and IRT models can provide interval measures, (3) no MTT model can provide interval measures. An outline of these three themes is presented in Table 6.

**Table 6:** Themes exposing the incompatible views concerning the ability to obtain interval measures using RMT and IRT metrics.

Only RMT	Both RMT and IRT	No MTT
*The theory of conjoint measurement provides the only analytical framework that could be invoked to evaluate whether the resulting scale could be said to have interval as opposed to ordinal or nominal properties*. *In practice*, *such a rationale has seldom been applied empirically*, *and generally hinges upon making an analogy between the Rasch model and a specific version of the theory of conjoint measurement known as additive conjoint measurement*. [40, p. 564] *For items and persons to be able to conjointly define this common interval (logit) scale*, *mathematical models need to specify that ICCs should never cross and be parallel* […]. *Only Rasch models satisfy the requirements of non-crossing and parallel ICCs*. [89, p. 290] *The main strength of this model [Rasch] is that it allows for testing if the simple summed raw score is a sufficient statistic (which cannot be done with other models) and also tests whether or not the data are consistent with the axioms of conjoint measurement*, *so providing a transformation to interval scaling*, *which also cannot be done with other models*. [90, p. 142]	*In fact*, *the relation between conjoint measurement and IRT is not limited to the Rasch model*; *it can be set up for quite a broad class of probabilistic latent variable models*. *The most general and precise treatment we know of in this context is given by Scheiblechner* [116]. [96, p. 110] *An alternative approach would be to consider polynomial conjoint measurement*. *This would be an important advance since it could be used to justify interval scale interpretations when items have varying discriminations*. [97, p. 16] *The IRT statistical methods generate empirical estimates of item difficulty and person ability that are log-transformed and converted to a true interval measure*, *thus eliminating the limitations associated with rank order*, *ordinal scales*. [98, p. S143]	*The theory of conjoint measurement describes a hierarchy of conditions—the so-called cancellation conditions directly diagnostic of the ordinal-quantitative distinction*. *However*, *in general*, *those applying IRTs show no inclination to employ this conceptual resource*. *Instead*, *the prevailing tendency is to evaluate an IRT via indices of goodness of fit that are not necessarily diagnostic of quantitative structure*. *Even in this potentially promising area of psychometrics*, *practitioners generally follow the time-honoured tradition of Stevens*, *begging the question that the attributes of interest are quantitative*, *instead of investigating it*. [104, p. 103] *That is*, *it is possible that attributes that psychometricians aspire to measure are heterogeneous orders*, *that is*, *non-measurable attributes*, *and this fact is not incompatible with observing statistical fit to IRT models*. *For example*, *Black et al*. [111], *Commons et al*. [112], *Kyngdon* [113], *and Kyngdon and Richards* [114] *all present tests in which the only discernable structure manifest in the item sets is ordinal and yet IRT models fit the relevant data*. [105, p. 6] *At present*, *the claim that psychometricians are able to measure psychological attributes on interval scales is a myth*, *and it will remain so until it is recognized that the possibility of attributes*, *such as abilities*, *being merely ordinal is one that must be seriously considered*. [106, p. 267]

*Abbreviations*: RMT, Rasch Measurement Theory; IRT, Item Response Theory; MTT, Modern Test Theory; ICC, Item Characteristic Curve.

The proponents of the first theme [40, 65, 89–95] argue that RMT models (as opposed to IRT models) test whether or not data are consistent with the axioms of Additive Conjoint Measurement (ACM). As ACM is the only analytical framework to prove if a metric is at the interval level, only RMT models can provide interval scaling. They also argue that if Item Characteristic Curves (ICCs) cross each other (and therefore the ordering of the items depends on the ability location) interval scales cannot be obtained.

As regards the second theme [96–103], an explanation on how interval scale can be obtained via an IRT model was not provided in many cases, but it was mostly taken for granted with statements such as *IRT generates estimates of item difficulty and person ability that are log-transformed and converted to a true interval measure* [98]. One case justified it via simulation [103], and very few mentioned the existence of Polynomial Conjoint Measurement theory as a means to justify interval scale interpretations when ICCs cross each other.

The rationale for the last theme [104–110] referred to the untested assumption made in MTT models that the attributes assessed are quantitative. The proponents of this view defend that this assumption needs to be tested to be able to measure attributes on interval scales. A way of testing it is employing directly the axioms of ACM, not by assessing fit to a MTT model. In fact, there is evidence that IRT and RMT models can fit ordinal data [111–114].

In any case, Briggs [115] expressed a pragmatic perspective concerning the quantity assumption stating that *until one can demonstrate empirically that a violation of the quantity assumption* (*i*.*e*., *the “‘pathology’” of psychometricians*, *to use Michell’s language*) *leads to significant practical consequences* […] *there will be little incentive to invest the time and effort into a research agenda focused on the discovery of psychological attributes that are measurable in a classical sense*.

## Discussion

In this study, we aimed to examine the use of the term ‘metric’ in health and social sciences’ literature, focusing on the interval scale implication of the term in MTT. By employing a Text Mining strategy, we were able to identify a number of different uses of ‘metric’ in MTT. As a noun, in many cases it can be considered as a synonym of ‘scale’. The qualifiers ‘good’, ‘more appropriate’, and ‘strongest possible’, of ‘metric’, encountered in some articles, suggest that not all metrics are equal, but that some metrics are *better* than others. The more a metric approaches the interval level (i.e., the more *equal* the differences between values are), the better it is. As an adjective, the mainly use of ‘metric’ referred to measurement or indicated quantification (interval or ratio level), as it is done in multidimensional scaling [5]. Based on the findings of the thematic analysis, three incompatible views were identified concerning whether interval measures can be obtained via MTT models: only RMT, both RMT and IRT models, or no MTT model can provide interval measures. Be that as it may, the term ‘interval scale’ was considerably more used in the RMT than in the IRT paradigm.

### Customized definitions of ‘metric’

Based on the findings of this review, there is not one formal definition of the term ‘metric’ (as a noun) in the psychometric sense. This has given rise to customized uses of the term, which can cause confusion, such as the definition of ‘common metric’ as *an IRT model*, *such as the GRM (Graded Response Model) or the GPCM (Generalized Partial Credit Model)*, *that comprises parameters of items from various measures*, *measuring a common variable* as appears in the http://www.common-metrics.org/ web page. A link to the web page was identified in one of the articles from our review [117].

Another use which can cause confusion is ‘mathematical metric’ with a meaning other than the formal mathematical definition of ‘metric’ (see S1 Appendix). In an article identified in this review, Cook et al. [83] stated that a mathematical metric can be defined as a system of units and numbers (see Table 3). This statement deviates from the formal mathematical definition of ‘metric’, where the distances among values are known, but they do not need to be equal between consecutive values (i.e., there is no need of a unit). Indeed, if for x,y∈R we define the *metric* *d* as $d\left(x,y\right)=\frac{\left|x-y\right|}{\left|x|+|y\right|}$ if *x*, *y* ≠ 0 and *d*(*x*, *y*) = 0 if *x* = *y* = 0, we have that, taking for instance the real numbers 1, 2, 3, the distance from 1 to 2 is not the same as from 2 to 3: 1/3 = *d*(1, 2) ≠ *d*(2, 3) = 1/5. Hence, there is no constant unit.

### Measurement

The question that remains is whether ‘units’ are needed to measure. Not all the proponents of the three themes identified in this review understood ‘measurement’ in the same way. The mainstream of the first and second themes (ability to obtain interval scales via (1) only Rasch or (2) all MTT models) seems to have a ‘realist’ conception of psychological attributes as described by Boorsboom and Mellenbergh [96]. The realists consider that *measurement consists in finding out people’s position on an attribute that exists quite independently of the measurement process*. After having transformed raw scores (which, according to them are ordinal and therefore have no constant unit) to interval scales via Rasch or IRT models, both perspectives refer sometimes to the ‘logit’ as the unit of their scale [100]. Nevertheless, Humphry [118] argues that in both cases this logit unit comes from a probabilistic model, and therefore a measurement unit can only exist by virtue of uncertainty and error. As opposed to physical units, the units derived from MTT models do not have an ontological status. Therefore, he suggests calling them ‘quasi-units’. Hence, there is no consensus as regards the existence of a unit in MTT scales, and no clear answer on whether it is needed to measure. In any case, according to the findings of our review, there is only one psychometric scale—the Lexile scale [63], which has a unit of measurement explicitly defined.

From their part, the proponents of the third theme (no MTT can provide interval scaling) rely on the classical definition of measurement (*a measurement is the product of a real number and a unit* [63]). Therefore, a unit is needed.

In contrast, according to the VIM (International Vocabulary of Metrology) [119] a unit is not needed. Indeed, measurement is defined as a *process of experimentally obtaining one or more quantity values that can reasonably be attributed to a quantity*. A quantity is a *property of a phenomenon*, *body*, *or substance*, *where the property has a magnitude that can be expressed as a number and a reference*. The reference can be not only a measurement unit, but also a measurement procedure, a reference material, or a combination of such. Finally, the VIM distinguishes an ordinal quantity as *quantity*, *defined by a conventional measurement procedure*, *for which a total ordering relation can be established*, *according to magnitude*, *with other quantities of the same kind*, *but for which no algebraic operations among those quantities exist*. An ordinal quantity could not be measured according to the classical definition, because it has no measurement units, and differences and ratios among ordinal quantities have no physical meaning.

Whether ordinal quantities can be measured or not, in any case they are more limited than interval scales, because algebraic operations cannot be performed on them. In contrast, a mean or a standard deviation can be computed on interval scales; therefore, when a metric is defined with a mean and standard deviation—such as a T-metric, formally this metric needs to be at the interval level. The proponents of the first and second themes—RMT and IRT metrics are at the interval level, would argue that a Rasch or an IRT metric offers more possibilities than the raw scores (which according to them are ordinal, but that was also a topic of discussion in the articles from our review; see, e.g., [99]) because more operations are allowed on them. Whether correct or not, RMT articles elaborated more the justification to obtain interval scales via a RMT model than IRT articles. Nevertheless, in the third theme it is criticized the common practice of MTT to justify interval scaling via indices of goodness of fit to RMT or IRT models. It is argued that fit to a MTT model should not be considered as evidence for interval scaling, and what should be investigated is whether psychological attributes actually possess a quantitative structure.

### Recommendations

- To avoid customized uses of ‘metric’, a formal definition of it in the psychometric sense should be determined. Probably the one describing more concretely ‘metric’ in this sense is *the numbers that the observed measures take on when describing individuals’ standings on the construct of interest* [120].
- Another option would be to do a parallelism of what it is done in VIM with the term ‘quantity’, namely to specify ‘ordinal metric’ when the metric is ordinal and not interval or ratio. We believe that when ‘metric’ is used alone it is indirectly assumed that this metric is at least at the interval level. In this way, if ‘ordinal metric’ is specified, it is clear that algebraic operations are not allowed. Although there is no consensus on whether Rasch and IRT metrics are interval, the authors should justify why they think that this is the case if they use ‘metric’ with no specification.
- A refinement to the four levels of scale established by Stevens [4] could be considered. As the term ‘scale’ is used to measure quantities, we believe that ‘nominal scale’ is not appropriate. Instead, nominal property, as suggested in the VIM, should be used. In addition, the term ‘ordered metric scale’ should be considered. This term was introduced in the mid-20th century [3] and it is still used [25, 63]. It refers to a metric between ordinal and interval levels where the magnitude of differences can be ordered. Therefore, we suggest the levels ordinal, ordered, interval, and ratio. In relation to the previous point, when ‘metric’ is used alone, it applies to the latter two categories. The first two should be compulsory adjectives to describe the nature of the metric.

### Strengths and limitations

The use of Text Mining strategies in conducting systematic literature searches and reviews is promising. It allowed us to automatically select the articles according to the refined criteria. The fact of providing a table with the previous and posterior context of the term ‘metric’ was extremely valuable to be able to collect the different uses of the term.

At the same time, it is important to stress that in the conversion from PDF to TXT, some words were not well identified, such as ‘metric’ being read as ‘menic’ in one case. If words as ‘psychometrics’ were written in the end of a sentence like ‘psycho-metrics’, the corresponding article was flagged as containing ‘metric’. Therefore, few false positives or false negatives were present, but we believe that in a very low proportion and with no major impact in our results.

We did not compare the efficacy of TM against manual extraction, and this could be considered in the future as a methodological study. Nevertheless, it would have been quite awkward to manually fill in an Excel file with 2635 articles placed in the rows, and for each article search whether it contained each of the six search terms, being careful that they did not only appear in the reference section, that Rasch refers to G. Rasch [12], that IRT did stand for Item Response Theory, and that ‘metric’ is not part of a proper name. Moreover, for each ‘metric’ use, the previous and posterior context should have been collected to discuss the different uses of the term. The corresponding automatically generated file via TM contains 4312 rows.

Another limitation of the present study is the year restriction in the systematic search. Although almost 2000 records were excluded for review, we believe that we have gathered the main uses and meanings of the term ‘metric’, as well as the main views concerning the ability to obtain interval scales via RMT and IRT. Effectively, our TM based strategy opens up possibilities to review a very large number of manuscripts. The approach is innovative, and the way we have undertaken it is an attempt to manage the very large number of manuscripts forthcoming. We made the assumption that relevant findings from papers published previous to 2001 would be cited in the 2001–2005 period, and that relevant findings from 2006–2010 would be cited in papers from 2011–2015. Although untested, Table 3, third column, second point, shows an example that supports our assumption: findings from articles from 2006 [113], 2008 [112], and 2011 [111] were cited in a 2012 paper [105] to support the hypothesis that fit to a MTT model should not be considered as evidence for interval scaling.

Also, the justification based on the increase in the number of records after 2001 is potentially biased, because the amount of papers published in the recent years has increased.

A further limitation is having restricted the review to MTT. Although it would have made sense to include other potential areas such as factor analysis, we wanted to focus on the existing dispute confronting RMT and IRT.

Finally, this study differs from the mainstream systematic search and reviews in the fact that its aim was putting some light on a terminology issue, namely the implications of the use of the term ‘metric’. We are not aware of any other study to have done something similar in the field of psychometrics.

## Supporting information

S1 Appendix Mathematical metric definition. (PDF)

S2 Appendix PRISMA guidelines. (PDF)

S3 Appendix Systematic search and review protocol. (PDF)

S4 Appendix Text mining strategy. (PDF)

S5 Appendix Guidelines for paradigm and type allocation of the included articles. (PDF)

S6 Appendix Dataset of included studies. (XLSX)

S7 Appendix Complete list of metric uses, synonyms and definitions. (XLSX)

## References

[1] Stiff WP. The British Pharmacopoeia, with Reference to Weights and Measures. BMJ. 1862;2(96):458–461. doi: 10.1136/bmj.2.96.458

[2] Knebelman MS. CONFORMAL GEOMETRY OF GENERALIZED METRIC SPACES. Proc Natl Acad Sci U S A. 1929;15(4):376–9. 16587487 10.1073/pnas.15.4.376 PMC522470

[3] Coombs CH. Psychological scaling without a unit of measurement. Psychol Rev. 1950;57(3):145–58. doi: 10.1037/h0060984 15417683 10.1037/h0060984

[4] Stevens SS. On the Theory of Scales of Measurement. Science. 1946;103(2684):677–680. doi: 10.1126/science.103.2684.677 20984256

[5] Takane Y, Young FW, de Leeuw J. Nonmetric individual differences multidimensional scaling: An alternating least squares method with optimal scaling features. Psychometrika. 1977;42(1):7–67. doi: 10.1007/BF02293745

[6] Stevens SS. Mathematics, measurement, and psychophysics In: Handbook of experimental psychology. New York: Wiley; 1951.

[7] Schuman EL, Brace CL. Metric and morphologic variations in the dentition of the Liberian chimpanzee; comparisons with anthropoid and human dentitions. Hum Biol. 1954;26(3):239–68. 13221314

[8] Crane PK, Narasimhalu K, Gibbons LE, Mungas DM, Haneuse S, Larson EB, et al Item response theory facilitated cocalibrating cognitive tests and reduced bias in estimated rates of decline. J Clin Epidemiol. 2008;61(10):1018–1027.e9. doi: 10.1016/j.jclinepi.2007.11.011 18455909 10.1016/j.jclinepi.2007.11.011 PMC2762121

[9] Kemmler G, Holzner B, Kopp M, Dünser M, Greil R, Hahn E, et al Multidimensional scaling as a tool for analysing quality of life data. Qual Life Res. 2002;11(3):223–233. doi: 10.1023/A:1015207400490 12074260 10.1023/a:1015207400490

[10] Arons AM, Krabbe PF. Quantification of health by scaling similarity judgments. PLoS One. 2014;9(2):e89091 doi: 10.1371/journal.pone.0089091 24586520 10.1371/journal.pone.0089091 PMC3931677

[11] Bai J, Di C, Xiao L, Evenson KR, LaCroix AZ, Crainiceanu CM, et al An Activity Index for Raw Accelerometry Data and Its Comparison with Other Activity Metrics. PLoS One. 2016;11(8):e0160644 doi: 10.1371/journal.pone.0160644 27513333 10.1371/journal.pone.0160644 PMC4981309

[12] Rasch G. Probabilistic Models for Some Intelligence and Attainment Tests. Danmarks Paedagogiske Institut; 1960.

[13] Hambleton RK, Swaminathan H, Rogers HJ. Fundamentals of Item Response Theory. SAGE Publications; 1991.

[14] Andrich D. Rating scales and Rasch measurement. Expert Rev Pharmacoecon Outcomes Res. 2011;11(5):571–85. doi: 10.1586/erp.11.59 21958102 10.1586/erp.11.59

[15] Petrillo J, Cano SJ, McLeod LD, Coon CD. Using Classical Test Theory, Item Response Theory, and Rasch Measurement Theory to Evaluate Patient-Reported Outcome Measures: A Comparison of Worked Examples. Value Health. 2015;18(1):25–34. doi: 10.1016/j.jval.2014.10.005 25595231 10.1016/j.jval.2014.10.005

[16] Kahler E, Rogausch A, Brunner E, Himmel W. A parametric analysis of ordinal quality-of-life data can lead to erroneous results. J Clin Epidemiol. 2008;61(5):475–480. doi: 10.1016/j.jclinepi.2007.05.019 18394541 10.1016/j.jclinepi.2007.05.019

[17] Doganay Erdogan B, Leung YY, Pohl C, Tennant A, Conaghan PG. Minimal Clinically Important Difference as Applied in Rheumatology: An OMERACT Rasch Working Group Systematic Review and Critique. J Rheumatol. 2016;43(1):194–202. doi: 10.3899/jrheum.141150 26034156 10.3899/jrheum.141150

[18] Grant MJ, Booth A. A typology of reviews: an analysis of 14 review types and associated methodologies. Health Info Libr J. 2009;26(2):91–108. doi: 10.1111/j.1471-1842.2009.00848.x 19490148 10.1111/j.1471-1842.2009.00848.x

[19] Moher D, Liberati A, Tetzlaff J, Altman DG, The PG. Preferred Reporting Items for Systematic Reviews and Meta-Analyses: The PRISMA Statement. PLoS Med. 2009;6(7):e1000097 doi: 10.1371/journal.pmed.1000097 19621072 10.1371/journal.pmed.1000097 PMC2707599

[20] Luce RD, Tukey JW. Simultaneous conjoint measurement: A new type of fundamental measurement. J Math Psychol. 1964;1(1):1–27. doi: 10.1016/0022-2496(64)90015-X

[21] Brogden HE. The rasch model, the law of comparative judgment and additive conjoint measurement. Psychometrika. 1977;42(4):631–634. doi: 10.1007/BF02295985

[22] Braun V, Clarke V. Successful qualitative research A practical guide for beginners. SAGE Publications Ltd; 2013.

[23] Robinson JP, Lubienski ST. The development of gender achievement gaps in mathematics and reading during elementary and middle school: Examining direct cognitive assessments and teacher ratings. Am Educ Res J. 2011;48(2):268–302. doi: 10.3102/0002831210372249

[24] Carvalho LF, Primi R, Meyer GJ. Application of the rasch model in measuring personality disorders. Trends Psychiatry Psychother. 2012;34(2):101–109. doi: 10.1590/S2237-60892012000200009 25922929 10.1590/s2237-60892012000200009

[25] Noventa S, Stefanutti L, Vidotto G. An analysis of item response theory and Rasch models based on the most probable distribution method. Psychometrika. 2014;79(3):377–402. doi: 10.1007/s11336-013-9348-y 25205004 10.1007/s11336-013-9348-y

[26] Krause MS. The data analytic implications of human psychology’s dimensions being ordinally scaled. Rev Gen Psychol. 2013;17(3):318–325. doi: 10.1037/a0032292

[27] Svensson E. Construction of a single global scale for multi-item assessments of the same variable. Statistics in Medicine. 2001;20(24):3831–3846. doi: 10.1002/sim.1148 11782037 10.1002/sim.1148

[28] Hartig J, Frey A, Nold G, Klieme E. An Application of Explanatory Item Response Modeling for Model-Based Proficiency Scaling. Educ Psychol Meas. 2012;72(4):665–686. doi: 10.1177/0013164411430707

[29] Bolt DM, Deng S, Lee S. IRT model misspecification and measurement of growth in vertical scaling. J Educ Meas. 2014;51(2):141–162. doi: 10.1111/jedm.12039

[30] Oude Voshaar MA, ten Klooster PM, Glas CA, Vonkeman HE, Taal E, Krishnan E, et al Calibration of the PROMIS physical function item bank in Dutch patients with rheumatoid arthritis. PLoS One. 2014;9(3):e92367 doi: 10.1371/journal.pone.0092367 24637885 10.1371/journal.pone.0092367 PMC3956923

[31] Stark S, Chernyshenko OS, Drasgow F. Examining the effects of differential item (functioning and differential) test functioning on selection decisions: When are statistically significant effects practically important? J Appl Psychol. 2004;89(3):497–508. doi: 10.1037/0021-9010.89.3.497 15161408 10.1037/0021-9010.89.3.497

[32] Carle AC, Blumberg SJ, Moore KA, Mbwana K. Advanced psychometric methods for developing and evaluating cut-point-based indicators. Child Indic Res. 2011;4(1):101–126. doi: 10.1007/s12187-010-9075-1

[33] Childs RA, Elgie S, Gadalla T, Traub R, Jaciw AP. IRT-linked standard errors of weighted composites. PARE. 2004;9(13).

[34] Ndosi M, Tennant A, Bergsten U, Kukkurainen ML, Machado P, De La Torre-Aboki J, et al Cross-cultural validation of the Educational Needs Assessment Tool in RA in 7 European countries. BMC Musculoskelet Disord. 2011;12 doi: 10.1186/1471-2474-12-110 21609481 10.1186/1471-2474-12-110 PMC3126763

[35] Behmke DA, Atwood CH. Implementation and assessment of Cognitive Load Theory (CLT) based questions in an electronic homework and testing system. Chem Educ Res Pract. 2013;14(3):247–256. doi: 10.1039/C3RP20153H

[36] Andrich D, Hagquist C. Real and Artificial Differential Item Functioning. J Educ Behav Stat. 2012;37(3):387–416. doi: 10.3102/1076998611411913 10.1177/0013164414534258 PMC5965593 29795818

[37] De Boeck P, Wilson M, Acton G. A Conceptual and Psychometric Framework for Distinguishing Categories and Dimensions. Psychol Rev. 2005;112(1):129–158. doi: 10.1037/0033-295X.112.1.129 15631591 10.1037/0033-295X.112.1.129

[38] Bjorner JB, Kosinski M, Ware JE Jr. Using item response theory to calibrate the Headache Impact Test (HIT^*TM*^) to the metric of traditional headache scales. Qual Life Res. 2003;12(8):981–1002. 14651417 10.1023/a:1026123400242

[39] Yu CH, Popp SEO. Test equating by common items and common subjects: Concepts and applications. PARE. 2005;10(4):1–19.

[40] Briggs DC, Domingue B. The Gains From Vertical Scaling. J Educ Behav Stat. 2013;38(6):551–576. doi: 10.3102/1076998613508317

[41] Demars CE. An analytic comparison of effect sizes for differential item functioning. Appl Meas Educ. 2011;24(3):189–209. doi: 10.1080/08957347.2011.580255

[42] Cella D, Choi S, Garcia S, Cook KF, Rosenbloom S, Lai JS, et al Setting standards for severity of common symptoms in oncology using the PROMIS item banks and expert judgment. Qual Life Res. 2014;23(10):2651–2661. doi: 10.1007/s11136-014-0732-6 24938431 10.1007/s11136-014-0732-6 PMC4710358

[43] Lovaglio PG, Monzani E. Health of the nation outcome scales evaluation in a community setting population. Qual Life Res. 2012;21(9):1643–1653. doi: 10.1007/s11136-011-0071-9 22120893 10.1007/s11136-011-0071-9

[44] Forero CG, Adroher ND, Stewart-Brown S, Castellví P, Codony M, Vilagut G, et al Differential item and test functioning methodology indicated that item response bias was not a substantial cause of country differences in mental well-being. J Clin Epidemiol. 2014;67(12):1364–1374. doi: 10.1016/j.jclinepi.2014.06.017 25150627 10.1016/j.jclinepi.2014.06.017

[45] Schalet BD, Rothrock NE, Hays RD, Kazis LE, Cook KF, Rutsohn JP, et al Linking Physical and Mental Health Summary Scores from the Veterans RAND 12-Item Health Survey (VR-12) to the PROMIS^®^ Global Health Scale. J Gen Intern Med. 2015;30(10):1524–1530. doi: 10.1007/s11606-015-3453-9 26179820 10.1007/s11606-015-3453-9 PMC4579239

[46] Hung M, Nickisch F, Beals TC, Greene T, Clegg DO, Saltzman CL. New paradigm for patient-reported outcomes assessment in foot & ankle research: Computerized adaptive testing. Foot Ankle Int. 2012;33(8):621–626. doi: 10.3113/FAI.2012.0621 22995227 10.3113/FAI.2012.0621

[47] Hays RD, Spritzer KL, Thompson WW, Cella D. U.S. General Population Estimate for “Excellent” to “Poor” Self-Rated Health Item. J Gen Intern Med. 2015;30(10):1511–1516. doi: 10.1007/s11606-015-3290-x 25832617 10.1007/s11606-015-3290-x PMC4579204

[48] Cohen AS, Kane MT, Seock-Ho K. The precision of simulation study results. Appl Psychol Meas. 2001;25(2):136–145. doi: 10.1177/01466210122031966

[49] Wahl I, Löwe B, Bjorner JB, Fischer F, Langs G, Voderholzer U, et al Standardization of depression measurement: A common metric was developed for 11 self-report depression measures. J Clin Epidemiol. 2014;67(1):73–86. doi: 10.1016/j.jclinepi.2013.04.019 24262771 10.1016/j.jclinepi.2013.04.019

[50] Golino HF, Gomes CMA, Commons ML, Miller PM. The construction and validation of a developmental test for stage identification: Two exploratory studies. Behav Dev Bull. 2014;19(3):37–54. doi: 10.1037/h0100589

[51] Andrews G, Halford GS. A cognitive complexity metric applied to cognitive development. Cogn Psychol. 2002;45(2):153–219. doi: 10.1016/S0010-0285(02)00002-6 12528901 10.1016/s0010-0285(02)00002-6

[52] Morse BJ, Johanson GA, Griffeth RW. Using the Graded Response Model to Control Spurious Interactions in Moderated Multiple Regression. Appl Psychol Meas. 2012;36(2):122–146. doi: 10.1177/0146621612438725

[53] Reise SP, Haviland MG. Item response theory and the measurement of clinical change. J Pers Assess. 2005;84(3):228–238. doi: 10.1207/s15327752jpa8403_02 15907159 10.1207/s15327752jpa8403_02

[54] Sudweeks RR, Reeve S, Bradshaw WS. A comparison of generalizability theory and many-facet Rasch measurement in an analysis of college sophomore writing. Assess Writ. 2004;9(3):239–261. doi: 10.1016/j.asw.2004.11.001

[55] Reise SP, Ainsworth AT, Haviland MG. Item response theory: Fundamentals, applications, and promise in psychological research. Curr Dir Psychol Sci. 2005;14(2):95–101. doi: 10.1111/j.0963-7214.2005.00342.x

[56] Smith RM, Taylor PA. Equating rehabilitation outcome scales: Developing common metrics. J Appl Meas. 2004;5(3):229–242. 15243171

[57] Perdices M. Some thoughts about the suitability of the reliable change index (RCI) for analysis of ordinal scale data. Brain Impair. 2015;15(3):223–232. doi: 10.1017/BrImp.2014.26

[58] Beltyukova SA, Stone GE, Fox CM. Equating Student Satisfaction Measures. J Appl Meas. 2004;5(1):62–69. 14757992

[59] Cieza A, Oberhauser C, Bickenbach J, Jones RN, Üstün TB, Kostanjsek N, et al The english are healthier than the Americans: Really? Int J Epidemiol. 2015;44(1):229–238. doi: 10.1093/ije/dyu182 25231371 10.1093/ije/dyu182 PMC4339758

[60] Dupuis M, Meier E, Capel R, Gendre F. Measuring individuals’ response quality in self-administered psychological tests: An introduction to Gendre’s functional method. Front Psychol. 2015;6(629). doi: 10.3389/fpsyg.2015.00629 26136693 10.3389/fpsyg.2015.00629 PMC4470441

[61] Dencker A, Sunnerhagen KS, Taft C, Lundgren-Nilsson A. Multidimensional fatigue inventory and post-polio syndrome—a Rasch analysis. Health Qual Life Outcomes. 2015;13(1). doi: 10.1186/s12955-015-0213-9 25879413 10.1186/s12955-015-0213-9 PMC4331415

[62] Humphry S. Item set discrimination and the unit in the Rasch model. J Appl Meas. 2012;13(2):165–180. 22805360

[63] Kyngdon A. Descriptive theories of behaviour may allow for the scientific measurement of psychological attributes. Theory Psychol. 2013;23(2):227–250. doi: 10.1177/0959354312468221

[64] Ewing MT, Salzberger T, Sinkovics RR. An alternate approach to assessing cross-cultural measurement equivalence in advertising research. J Advert. 2005;34(1):17–36. doi: 10.1080/00913367.2005.10639181

[65] Sijtsma K. Psychological measurement between physics and statistics. Theory Psychol. 2012;22(6):786–809. doi: 10.1177/0959354312454353

[66] Pae HK. A psychometric measurement model for adult English language learners: Pearson Test of English Academic. Educ Res Eval. 2012;18(3):211–229. doi: 10.1080/13803611.2011.650921

[67] Hadzibajramovic E, Ahlborg G, Hakansson C, Lundgren Nilsson A, Grimby Ekman A. Affective stress responses during leisure time: Validity evaluation of a modified version of the Stress-Energy Questionnaire. Scand J Public Health. 2015;43(8):825–832. doi: 10.1177/1403494815601552 26392419 10.1177/1403494815601552

[68] Oreja-Rodríguez JR, Armas-Cruz Y. Environmental performance in the hotel sector: The case of the Western Canary Islands. J Clean Prod. 2012;29–30:64–72. doi: 10.1016/j.jclepro.2012.02.012

[69] Küçükdeveci AA, Tennant A, Grimby G, Franchignoni F. Strategies for assessment and outcome measurement in physical and rehabilitation medicine: An educational review. J Rehabil Med. 2011;43(8):661–672. doi: 10.2340/16501977-0844 21687922 10.2340/16501977-0844

[70] Arnould C, VanDervelDe L, Batcho CS, Penta M, Thonnard JL. Can manual ability be measured with a generic ABILHAND scale? A cross-sectional study conducted on six diagnostic groups. BMJ Open. 2012;2(6). doi: 10.1136/bmjopen-2012-001807 23117570 10.1136/bmjopen-2012-001807 PMC3533037

[71] Ødegaard F, Roos P. Measuring the contribution of workers’ health and psychosocial work-environment on production efficiency. Prod Oper Manag. 2014;23(12):2191–2208. doi: 10.1111/poms.12242

[72] Sanchez-Garcia M, Fernandez-Calderon F, Carmona-Marquez J, Chico-Garcia M, Velez-Moreno A, Perez-Gomez L. Psychometric properties and adaptation of the ASRS in a Spanish sample of patients with substance use disorders: Application of two IRT rasch models. Psychol Assess. 2015;27(2):524–533. doi: 10.1037/pas0000064 25580610 10.1037/pas0000064

[73] Vilagut G, Forero CG, Adroher ND, Olariu E, Cella D, Alonso J, et al Testing the PROMIS^®^ Depression measures for monitoring depression in a clinical sample outside the US. J Psychiatr Res. 2015;68:140–150. doi: 10.1016/j.jpsychires.2015.06.009 26228413 10.1016/j.jpsychires.2015.06.009

[74] Miguel JP, Silva JT, Prieto G. Career Decision Self-Efficacy Scale—Short Form: A Rasch analysis of the Portuguese version. J Vocat Behav. 2013;82(2):116–123. doi: 10.1016/j.jvb.2012.12.001

[75] Lopes P, Prieto G, Delgado AR. A Rasch analysis of the harm reduction self-efficacy questionnaire in Portugal. Addict Behav. 2014;39(10):1500–1503. doi: 10.1016/j.addbeh.2014.05.014 24954444 10.1016/j.addbeh.2014.05.014

[76] Simone A, Rota V, Tesio L, Perucca L. Generic ABILHAND questionnaire can measure manual ability across a variety of motor impairments. Int J Rehabil Res. 2011;34(2):131–140. doi: 10.1097/MRR.0b013e328343d4d3 21383629 10.1097/MRR.0b013e328343d4d3

[77] La Porta F, Franceschini M, Caselli S, Cavallini P, Susassi S, Tennant A. Unified Balance Scale: An activity-based, bed to community, and Aetiology-in dependent measure of balance calibrated with Rasch analysis. J Rehabil Med. 2011;43(5):435–444. doi: 10.2340/16501977-0797 21394420 10.2340/16501977-0797

[78] Bakhsh H, Franchignoni F, Ferriero G, Giordano A, Demers L. Translation into Arabic of the Quebec User Evaluation of Satisfaction with Assistive Technology 2.0 and validation in orthosis users. Int J Rehabil Res. 2014;37(4):361–367. doi: 10.1097/MRR.0000000000000086 25305007 10.1097/MRR.0000000000000086

[79] Tang K, Beaton DE, Gignac MAM, Bombardier C. Rasch analysis informed modifications to the Work Instability Scale for Rheumatoid Arthritis for use in work-related upper limb disorders. J Clin Epidemiol. 2011;64(11):1242–1251. doi: 10.1016/j.jclinepi.2011.02.002 21530170 10.1016/j.jclinepi.2011.02.002

[80] DeMars CE, Jurich DP. The Interaction of Ability Differences and Guessing When Modeling Differential Item Functioning With the Rasch Model: Conventional and Tailored Calibration. Educ Psychol Meas. 2015;75(4):610–633. doi: 10.1177/0013164414554082 10.1177/0013164414554082 PMC5965617 29795835

[81] Tulsky DS, Kisala PA, Victorson D, Choi SW, Gershon R, Heinemann AW, et al Methodology for the development and calibration of the SCI-QOL item banks. J Spinal Cord Med. 2015;38(3):270–287. doi: 10.1179/2045772315Y.0000000034 26010963 10.1179/2045772315Y.0000000034 PMC4445019

[82] Kyngdon A. Psychological measurement needs units, ratios, and real quantities: A commentary on Humphry. Measurement. 2011;9(1):55–58.

[83] Cook KF, O’Malley KJ, Roddey TS. Dynamic assessment of health outcomes: Time to let the CAT out of the bag? Health Serv Res. 2005;40(5 II):1694–1711. doi: 10.1111/j.1475-6773.2005.00446.x 16179003 10.1111/j.1475-6773.2005.00446.x PMC1361218

[84] Bedell GM. Developing a follow-up survey focused on participation of children and youth with acquired brain injuries after discharge from inpatient rehabilitation. NeuroRehabilitation. 2004;19(3):191–205. 15502253

[85] Yusoff R, Mohd Janor R. Generation of an interval metric scale to measure attitude. SAGE Open. 2014;4(1). doi: 10.1177/2158244013516768

[86] Cieza A, Sabariego C, Anczewska M, Ballert C, Bickenbach J, Cabello M, et al PARADISE 24: A measure to assess the impact of brain disorders on people’s lives. PLoS One. 2015;10(7). doi: 10.1371/journal.pone.0132410 10.1371/journal.pone.0132410 PMC4492620 26147343

[87] Betemps EJ, Smith RM, Baker DG, Rounds-Kugler BA. Measurement Precision of the Clinician Administered PTSD Scale (CAPS): A RASCH Model Analysis. J Appl Meas. 2003;4(1):59–69. 12700431

[88] Penfield RD. The Impact of Model Misfit on Partial Credit Model Parameter Estimates. J Appl Meas. 2004;5(2):115–128. 15064532

[89] Smith J Everett V. Evidence for the reliability of measures and validity of measure interpretation: A Rasch measurement perspective. J Appl Meas. 2001;2(3):281–311. 12011511

[90] Da Rocha NS, Chachamovich E, de Almeida Fleck MP, Tennant A. An introduction to Rasch analysis for Psychiatric practice and research. J Psychiatr Res. 2013;47(2):141–148. doi: 10.1016/j.jpsychires.2012.09.014 23069651 10.1016/j.jpsychires.2012.09.014

[91] Siemons L, Krishnan E. A short tutorial on item response theory in rheumatology. Clin Exp Rheumatol. 2014;32(4):581–586. 25065775

[92] Reise SP, Henson JM. A discussion of modern versus traditional psychometrics as applied to personality assessment scales. J Pers Assess. 2003;81(2):93–103. doi: 10.1207/S15327752JPA8102_01 12946916 10.1207/S15327752JPA8102_01

[93] Andrich D. Controversy and the Rasch model: a characteristic of incompatible paradigms? Med Care. 2004;42(1 Suppl). 14707751 10.1097/01.mlr.0000103528.48582.7c

[94] Fisher WP. Mathematics, Measurement, Metaphor and Metaphysics II: Accounting for Galileo’s ‘Fateful Omission’. Theory Psychol. 2003;13(6):791–828. doi: 10.1177/0959354303136003

[95] Salzberger T. Attempting measurement of psychological attributes. Front Psychol. 2013;4(FEB). doi: 10.3389/fpsyg.2013.00075 23550264 10.3389/fpsyg.2013.00075 PMC3581806

[96] Borsboom D, Mellenbergh GJ. Why Psychometrics is Not Pathological: A Comment on Michell. Theory Psychol. 2004;14(1):105–120. doi: 10.1177/0959354304040200

[97] Domingue B. Evaluating the Equal-Interval Hypothesis with Test Score Scales. Psychometrika. 2014;79(1):1–19. doi: 10.1007/s11336-013-9342-4 24532164 10.1007/s11336-013-9342-4

[98] Seel RT, Steyerberg EW, Malec JF, Sherer M, MacCiocchi SN. Developing and evaluating prediction models in rehabilitation populations. Arch Phys Med Rehabil. 2012;93(8 SUPPL.). 10.1016/j.apmr.2012.04.021 22840880

[99] Lovelace M, Brickman P. Best practices for measuring students’ attitudes toward learning science. CBE Life Sci Educ. 2013;12(4):606–617. doi: 10.1187/cbe.12-11-0197 24297288 10.1187/cbe.12-11-0197 PMC3846512

[100] Massof RW. Understanding rasch and item response theory models: Applications to the estimation and validation of interval latent trait measures from responses to rating scale questionnaires. Ophthalmic Epidemiol. 2011;18(1):1–19. doi: 10.3109/09286586.2010.545501 21275592 10.3109/09286586.2010.545501

[101] Vanhoutte EK, Hermans MCE, Faber CG, Gorson KC, Merkies ISJ, Thonnard JL. Rasch-ionale for neurologists. J Peripher Nerv Syst. 2015;20(3):260–268. doi: 10.1111/jns.12122 26115370 10.1111/jns.12122

[102] Reeve BB. Item response theory modeling in health outcomes measurement. Expert Rev Pharmacoecon Outcomes Res. 2003;3(2):131–145. doi: 10.1586/14737167.3.2.131 19807361 10.1586/14737167.3.2.131

[103] Harwell MR, Gatti GG. Rescaling ordinal data to interval data in educational research. Rev Educ Res. 2001;71(1):105–131. doi: 10.3102/00346543071001105

[104] Michell J. Stevens’s theory of scales of measurement and its place in modern psychology. Aust J Psychol. 2002;54(2):99–104. doi: 10.1080/00049530210001706563

[105] Michell J. Alfred Binet and the concept of heterogeneous orders. Front Psychol. 2012;3:261 doi: 10.3389/fpsyg.2012.00261 22912619 10.3389/fpsyg.2012.00261 PMC3419461

[106] Michell J. “The constantly recurring argument”: Inferring quantity from order. Theory Psychol. 2012;22(3):255–271. doi: 10.1177/0959354311434656

[107] Heene M. Additive conjoint measurement and the resistance toward falsifiability in psychology. Front Psychol. 2013;4:246 doi: 10.3389/fpsyg.2013.00246 23653615 10.3389/fpsyg.2013.00246 PMC3644681

[108] McGrane JA. Stevens’ forgotten crossroads: The divergent measurement traditions in the physical and psychological sciences from the mid-twentieth century. Front Psychol. 2015;6(MAR).

[109] McGrath RE. Rethinking psychosocial constructs: Reply to comments by Barrett, Kagan, and Maraun and Peters. J Pers Assess. 2005;85(2):141–145. doi: 10.1207/s15327752jpa8502_06

[110] Michell J. Measurement: A Beginner’s Guide. J Appl Meas. 2003;4(4):298–308. 14523251

[111] Black P, Wilson M, Yao SY. Road Maps for Learning: A Guide to the Navigation of Learning Progressions. Measurement. 2011;9(2–3):71–123.

[112] Commons ML, Goodheart EA, Pekker A, Dawson TL, Draney K, Adams KM. Using Rasch scaled stage scores to validate orders of hierarchical complexity of balance beam task sequences. J Appl Meas. 2008;9(2):182–99. 18480514

[113] Kyngdon A. An empirical study into the theory of unidimensional unfolding. J Appl Meas. 2006;7(4):369–93. 17068378

[114] Kyngdon A, Richards B. Attitudes, order and quantity: deterministic and direct probabilistic tests of unidimensional unfolding. J Appl Meas. 2007;8(1):1–34. 17215563

[115] Briggs DC. Measuring growth with vertical scales. J Educ Meas. 2013;50(2):204–226. doi: 10.1111/jedm.12011

[116] Scheiblechner H. Additive conjoint isotonic probabilistic models (ADISOP). Psychometrika. 1999;64(3):295–316. doi: 10.1007/BF02294297

[117] Liegl G, Wahl I, Berghofer A, Nolte S, Pieh C, Rose M, et al Using Patient Health Questionnaire-9 item parameters of a common metric resulted in similar depression scores compared to independent item response theory model reestimation. J Clin Epidemiol. 2016;71:25–34. doi: 10.1016/j.jclinepi.2015.10.006 26475569 10.1016/j.jclinepi.2015.10.006

[118] Humphry SM. A middle path between abandoning measurement and measurement theory. Theory Psychol. 2013;23(6):770–785. doi: 10.1177/0959354313499638

[119] Bureau International des Poids et Mesures. International vocabulary of metrology—Basic and general concepts and associated terms (VIM); 2008. http://www.bipm.org/utils/common/documents/jcgm/JCGM_200_2012.pdf.

[120] Blanton H, Jaccard J. Arbitrary metrics in psychology. Am Psychol. 2006;61(1):27–41. doi: 10.1037/0003-066X.61.1.62 16435974 10.1037/0003-066X.61.1.27

