# Supplementary material for: All metrics are equal, but some metrics are more equal than others: A systematic search and review on the use of the term ‘metric’
Source: PLoS One. 2018 Mar 6;13(3):e0193861. doi: 10.1371/journal.pone.0193861 (PMC5839589; doi:10.1371/journal.pone.0193861)
Supplement: S1 Appendix — (PDF) [file pone.0193861.s001.pdf]

Mathematical metric definition

A metric space consists of a pair (X, d), where X is a set and $d : X \times X \to \mathbb{R}^+$ is a function, called the metric or distance function, such that for all $x, y, z \in X$

1. (Symmetry) $d(x, y) = d(y, x)$
2. (Positive Definiteness) $d(x, y) \geq 0$, and $d(x, y) = 0$ if and only if $x = y$
3. (Triangle Inequality) $d(x, z) \leq d(x, y) + d(y, z)$
