# Supplementary material for: All metrics are equal, but some metrics are more equal than others: A systematic search and review on the use of the term ‘metric’
Source: PLoS One. 2018 Mar 6;13(3):e0193861. doi: 10.1371/journal.pone.0193861 (PMC5839589; doi:10.1371/journal.pone.0193861)
Supplement: S3 Appendix — (PDF) [file pone.0193861.s003.pdf]

Systematic Search and Review Methodology

As explained in Grant et al. [1], a Systematic Search and Review combines the strengths of a critical review with a comprehensive search process. Typically, this type of review addresses broad questions and the result is a 'best evidence synthesis'.

Protocol

Purpose of the study:
To examine the use of the term 'metric' in health and social sciences' literature, focusing on the interval scale implication of the term in Modern Test Theory (MTT).

- Aim 1: To explore the different uses and meanings of 'metric' in MTT literature.
- Aim 2: To examine the relationships between 'metric' and 'interval scale' in Rasch Measurement Theory (RMT) and Item Response Theory (IRT).
- Aim 3: To compare the current understanding on whether either or both paradigms can produce interval scaling.

How to conduct the review to respond to the purpose of the study

Method	Description	Application in our study
Search	Aims for exhaustive, comprehensive searching	**Databases**:<br>SCOPUS, PsychINFO, PubMed and ERIC to gather evidence from the health, social, psychological, behavioral, and educational sciences.<br><br>**Search terms**:<br>#C1 (Rasch OR item response OR IRT)<br>AND<br>#C2 (metric OR interval scale OR conjoint measurement OR fundamental measurement)<br><br>C1 identified articles from the MTT field.<br>C2 contained 'metric' to respond to Aim 1, 'interval scale' to respond to Aims 2 and 3, and 'conjoint measurement' and 'fundamental measurement' to respond to Aim 3.<br><br>**Inclusion/Exclusion criteria**<br>I1: Article published in English in a peer-reviewed journal.<br>I2: Full text of the article containing at least one C1 term and one C2 term.<br><br>E1: Article published in a journal out of the health and social sciences.<br>E2: Terms in C1 or in C2 appearing only in the reference section.<br>E3: Only Rasch was mentioned in C1, and it did not refer to the Rasch model or to Georg Rasch.<br>E4: Only IRT was mentioned in C1, and it referred to an acronym other than Item Response Theory.<br>E5: Only 'metric' was mentioned in C2, and it was part of a proper name.<br><br>**Year restriction**<br>The search was restricted to 2001-2005, 2011-2015 due to the vast amount of records identified in the database search. We hypothesized that relevant findings from papers published previous to 2001 would be cited in 2001-2005, and relevant findings from papers published in 2006-2010 would be cited in 2011-2015.

Appraisal	May or may not include quality assessment	Where appropriate, the review followed the PRISMA guidelines for systematic reviews. I1 and E1 were checked manually, and I2 and E2-E5 and via a Text Mining (TM) strategy. The TM algorithm is described in the supplementary materials of the manuscript of the study. Quality assessment in this study implies checking whether I/E criteria were correctly fulfilled, and that was done during the implementation of the TM strategy.
Synthesis	Minimal narrative, tabular summary of studies	Each article was assigned to a Paradigm (RMT, IRT, or MTT) to respond to Aim 2, and to a Type (Theoretical, Methodological, Teaching, Application, Miscellaneous) to respond to Aim 3.<br>An Excel file with the included articles in rows, and for each of them, the corresponding Paradigm, Type, number of uses of each of the search terms, and inclusion for Full text review, is available in the supplementary materials of the article.<br>The uses of 'metric' were collected in a table distinguishing the parts of the speech noun, adjective or adverb. Synonyms and definitions of 'metric' were also considered. The relationship of 'metric' and 'interval scale' was explored via contingency tables. The full text of Theoretical, Methodological, and Teaching articles was read to compare the understanding on whether either or both paradigms can produce interval scales.
Analysis	What is known; recommendations for practice. Limitations	The tables described in "Synthesis" were examined.<br>An inductive thematic analysis was performed on the Theoretical, Methodological, and Teaching articles. Passages from the articles related to Aim 3 were extracted to a Word document, and different themes were identified.<br>Recommendations, strengths, and limitations of the study were listed in the Discussion section of the manuscript.

Table S3.1: Methodology of the systematic search and review as described in Grant et al. [1].

1. Grant, M.J. and A. Booth, *A typology of reviews: an analysis of 14 review types and associated methodologies.* Health Info Libr J, 2009. **26**(2): p. 91-108.
