# Supplementary material for: All metrics are equal, but some metrics are more equal than others: A systematic search and review on the use of the term ‘metric’
Source: PLoS One. 2018 Mar 6;13(3):e0193861. doi: 10.1371/journal.pone.0193861 (PMC5839589; doi:10.1371/journal.pone.0193861)
Supplement: S4 Appendix — (PDF) [file pone.0193861.s004.pdf]

Text Mining strategy

Algorithm 1 Article eligibility

1. Convert the articles from PDF to TXT format using the **Corpus** function from the R **tm** package.
2. Create a Corpus (Corp1) including all the TXTs using the **VCorpus** function from **tm**.
3. Perform a quality check to Corp1 to ensure that all the PDFs were correctly converted to TXTs, and correct potential issues.
4. Create a new Corpus (Corp2) from Corp1 containing the articles without the references, so that E2 (Exclusion criterion 2) is fulfilled. E2: Terms in Combination 1 [Rasch or IRT or item response] or in Combination 2 [metric or interval scale or conjoint measurement or fundamental measurement] appear only in the reference section.
5. Remove the following punctuation symbols in Corp2 [](){}*=¿?.;:,¡!·` ”, and substitute - and ’ with a blank space.
6. Apply the **kwic** function from the R **quanteda** package to each of the terms in Table S4.1. This function lists the term as well as its previous and post context of instance in each of the articles. In this way we can identify the papers in which each of the terms appeared. Table S4.2 shows some articles including the term ‘metric’.
7. Create the FRET matrix (Table S4.3) which contains, for each article, the frequency of instances of each of the terms in Combination 1 and Combination 2.
8. Add 3 binary columns to FRET: the first indicates if the article contains any of the terms in Combination 1, the second if the article contains any of the terms in Combination 2, and the third indicates if the sum of the previous columns is 2 (namely, if the article is included).
9. Perform a quality check of the final eligibility decision:
    - Take a random sample of the included and excluded articles and manually check if they were correctly included or excluded.
    - Check E3: Identify ‘Rasch’ not referring to the Rasch model or to Georg Rasch.
    - Check E4: Identify other IRT meanings.
    - Check E5: Identify proper names containing ‘metric’.
    - Identify articles with one or two instances of Rasch and IRT and manually check if the instances really correspond to the Rasch model or IRT methods (through this way other Rasch authors or other IRT meanings were found). Some articles were incorrectly included because Rasch/IRT was only mentioned at the beginning of the References section, and it incorrectly appeared before the Reference section when transformed to TXT).
10. Correct the FRET matrix, excluding the articles identified in the previous point.

Table S4.1. Alternatives for the terms from Combinations 1 and 2

Rasch	IRT	Metric	Interval scale	Conjoint Measurement	Fundamental Measurement
rasch	irt	metric	conjoint interval	conjoint measur*	fundamental measur*
SLM	item response analys*	metrica*	equal interval		
PRM	item response method*	metrici*	equalinterval		
MFRM	item response model*	metrics	equated interval		
RSM	item response theory		interval based		
PCM	1PL		interval characteristics		
	2PL		interval data		
	3PL		interval item		
	4PL		interval level		
	GRM		interval logit		
	NRM		interval measur*		
	mixIRT		interval metric		
	Mokken		interval properties		
	GPCM		interval scal*		
	generalized PCM		interval scor*		
	generalised PCM		interval units		
			interval variable		
			intervale scale		
			intervalist		
			intervallevel		
			intervally scaled		
			intervalness		
			ordinal to interval		
			quasi interval		

Abbreviations: IRT, Item Response Theory; SLM, Simple Logistic Model; PRM, Polytomous Rasch Model; MFRM, Many-Facet Rasch Model; RSM, Rating Scale Model; PCM, Partial Credit Model; 1P, one-parameter model; 2P, two-parameter model; 3P, three-parameter model; 4P, four-parameter model; GRM, Graded Response Model; NRM, Nominal Response Model; GPCM, Generalized Partial Credit Model.

Table S4.2. Some uses of 'metric'

Article	Previous context	keyword	Posterior context
Abad_2004_PID	one needs to equate the	metrics	The linear coefficients were computed
AbdElFattah_2014_AJEDP	they are on the same	metric	Bond& Fox 2007 The
AbdElFattah_2014_AJEDP	The Rasch model assesses the	metric	properties of unidimensionality and provides
AbdElFattah_2014_AJEDP	be measured using the same	metric	and placed on the same

Table S4.3. FRET matrix, Frequency of search terms by articles to determine the included articles

Article	RMT	IRT	Metric	Interval scale	Conjoint Measurement	Fundamental Measurement	C1	C2	Inclusion
Aabenhus_2013_ViH	21	0	0	0	0	0	1	0	0
Abad_2004_PID	1	9	3	0	0	0	1	1	1
Abbas_2014_BMCMedIm	0	6	0	0	0	0	1	0	0
Abbatiello_2013_MolCelPro	0	0	35	0	0	0	0	1	0
Abdallah_2015_JTAER	0	0	1	2	0	0	0	1	0
AbdElFattah_2014_AJEDP	34	1	3	1	0	0	1	1	1

Abbreviations: RMT, Rasch Measurement Theory; IRT, Item Response Theory; C, Combination. In the C1 column, 1 indicates that article contains any of the terms in Combination 1, and 0 that it does not. The same applies to C2 and combination 2. The Inclusion column indicates if the sum of the previous columns is 2 (namely, if the article is included).
