# Supplementary material for: All metrics are equal, but some metrics are more equal than others: A systematic search and review on the use of the term ‘metric’
Source: PLoS One. 2018 Mar 6;13(3):e0193861. doi: 10.1371/journal.pone.0193861 (PMC5839589; doi:10.1371/journal.pone.0193861)
Supplement: S5 Appendix — (PDF) [file pone.0193861.s005.pdf]

Guidelines for Paradigm and Type Allocation of the included articles

Paradigm Allocation

Not only the mere application of RMT or IRT models should be taken into account for the paradigm allocation, but if the paper considers or distinguishes one or both paradigms as well.

If the information in the abstract does not suffice to allocate the paradigm, search Rasch/Item response/IRT using CTRL+SHIFT+F through the full text to determine the paradigm.

- RMT. If Rasch is described as an IRT model, but only Rasch is applied, and there is no distinction between RMT/IRT, then the Paradigm is RMT, because RMT is predominant versus IRT. Examples of papers belonging to this type are presented:
  - Alderman_2012_NeupsyhReh: *Use of CTT methods in instances like this makes a number of assumptions. Item response theory (IRT) provides a range of mathematical models that test these. The model developed by Rasch (1960) ...*
  - Baghaei_2014_SAGEopen: *Furthermore, unlike two-parameter and three -parameter logistic IRT models, no assumptions regarding the normality of the distribution of person traits need to be made. These primary advantages drove the decision to use the Rasch model in the present study to evaluate the psychometric properties of the CTAR-17..*
  - Cadime_2014_AnaPsy: *The results of the model fit also provide support for our option of using the Rasch model instead of a more complex one (e.g., a two or three parameter IRT model) in the parameters estimation. According to the parsimony principle, when we have more than one model with good fit to the data, the simpler model should be preferred.*
  - Dunn_2014_AJOT: *The Rasch model was applied to conduct a more detailed item analysis to examine the structure of the CHORES subscales.The Rasch model derives from item response theory and has been used in the construction and validation of several rehabilitation- and healthrelated measures.*
  - Twiss_2015_QoLR: *Scales that do not fit the Rasch model cannot be co-calibrated in the way described in this study using Rasch analysis. Other, less restrictive, item response theory models are also available for co-calibrating measures.*
  - Yazdani_2015_IJPH: *Problems that can result from this have led to the use of modern approaches, including Item Response Theory (IRT) and Rasch analysis that claims to be able to achieve fundamental measurement... According to the Likert type questions and based on Linacre's recommendations about choosing one of the polytomous models to analyze, the Rating Scale Analysis (RSA) was selected.*
- IRT. If Rasch is not mentioned, or if Rasch is mentioned among other IRT models (but not applied), and there is no distinction between RMT/IRT, then the paradigm is IRT. IRT is predominant versus RMT (if Rasch is mentioned as application in other paper, but the present one clearly focus on IRT, the paradigm is IRT). Examples:
  - Bolt_2014_JEdumea: *Most attention related to functional form misspecification has attended to the effects of fitting one traditional IRT*

model when the data are generated from another such model (e.g., Rasch, 2PL or 3PL).

  - Liegl_2016_JCE: *In contrast to models from the Rasch family [61,62], which are widely used for analyzing items with polytomous response format as well, GPCMs and GRMs do not require equal discrimination parameters for all included items and therefore seem to be more appropriate for analyzing PRO data.*

- MTT. The article compares/distinguishes RMT/IRT (applying models from both paradigms), or it applies Rasch within the IRT framework, mentioning IRT several times, not only presenting Rasch as an IRT model. IRT also takes an important part in the paper, even if at the end only Rasch is applied. If Rasch is only mentioned as example or listed, then it goes to IRT. Rasch and IRT have a similar presence.

  - Andrich_2004_MedCar: *Originally, there was substantial controversy between those who saw Rasch models as simply special cases of IRT models and those who saw them as essentially different.*
  - BondTN_2013_REcoSta: *In the special case where all questions are equally informative, a can be normalized to 1, which gives the one-parameter IRT model or Rasch scale.*
  - Dadey_2012_PARE: *The solid dots represent states using the 3PL/GPCM to calibrate their vertical scale; the empty dots represent states that used the Rasch Model/Partial Credit Model.*

Type Allocation

1. Theoretical. Main focus a theoretical issue involving RMT/IRT and measurement, written in formal or mathematical discourse. Philosophical discussions, as well as commentaries on other papers, are also included. If an application is presented, it should be as an example to clarify the discussed issue.

   - Krause_2013_RGenPsy: *So descriptions of these phenomena locate them in merely ordinal hyperspaces, which imposes severe constraints on data analysis for inducing or testing explanatory theory involving them. Therefore, it is important to be clear about what these constraints are and so what properly can be concluded on the basis of ordinal-scale multivariate data, which also provides a test for methods that are proposed to transform ordinal-scale data into ratio-scale data (e.g., classical test theory, item response theory, additive conjoint measurement), because such transformations must not violate these constraints and so distort descriptions of studied phenomena.*
   - Cantley_2015_TaP: *This article gives a brief, critical overview of the evolution of current measurement practices in psychology, and suggests the need for a transition from a Newtonian to a quantum theoretical paradigm for psychological measurement.*
   - BarrettP_2003_JManPsy: *By describing the key features of quantitative measurement, and contrasting these with current psychometric practice, it is argued that Michell is correct in his assertion.[...] It is to be hoped that psychometrics begins to concern itself more with the logic of its measurement, rather than the ever-increasing complexity of its numerical and statistical operations.*

- Michell_2012_TaP_B: *However, it might still be urged that the inference from order to quantity is an inference to the best explanation: that is, that quantitative structure is reasonably abduced from order. I argue that the opposite is true: the most plausible hypothesis is that the sorts of attributes psychometricians aspire to measure are merely ordinal attributes with impure differences of degree, a feature logically incompatible with quantitative structure.*
- Borsboom_2004_TaP: *This paper comments on an article by Michell (2000), who argues that psychometrics should be qualified as pathological science for two reasons.*
- FisherWPJ_2011_Measur_C: *Humphry's article, "'The Role of the Unit in Physics and Psychometrics,"' offers fundamental clarifications of measurement concepts that I hope will find a wide audience.*

2. Methodological: Not purely an applied paper because it identifies a weakness/something unknown in current methods which can be improved using RMT/IRT. A novel method can also be presented. Sometimes it compares different methods/models, via simulation or not. It focuses on both applied and theoretical aspects, not only theoretical. A property can be verified empirically. It does not include papers dealing with psychometric problems of specific questionnaires, neither papers dealing with theoretical aspects other than measurement.

    - Cipriani_2005_JAM: *These probability estimates correlated with the estimates of Sn, Sp, and LR. The RMM estimates were not affected by missing data. Discussion: The RMM may provide an alternative means to study the utility of medical diagnostic tests to estimate the probability of disease presence/absence.*
    - Hastedt_2015_PARE: *This paper endeavors to shed some light on the effects that can be expected, the linkage errors in particular, by countries using this practice.*
    - ChoSJ_2014_SEM: *The presence of nuisance dimensionality is a potential threat to the accuracy of results for tests calibrated using a measurement model such as a factor analytic model or an item response theory model. This article describes a mixture group bifactor model to account for the nuisance dimensionality due to a testlet structure as well as the dimensionality due to differences in patterns of responses.*
    - Garrard_2015_BMCMRM: *We propose an innovative Ordinal Bayesian Instrument Development (OBID) method that seamlessly integrates expert and participant data in a Bayesian item response theory (IRT) with a probit link model framework.*
    - Dupuis_2015_FiP: *The functional method is a new test theory using a new scoring method that assumes complexity in test structure, and thus takes into account every correlation between factors and items.*
    - CaoM_2015_ORM: *Among four different wording domains (i.e., specifying frequencies, comparing to average, specifying conditions, and using transitions) to create intermediate items, which method(s) will perform well in practice?*
    - KingJ_2003_JAM: *The Rasch modeling property of specific objectivity was empirically verified when calculation of identical benchmark estimates resulted from the construction of simulated population proportional samples using sample:population size weightings.*

3. Teaching: Teaching paper on RMT/IRT, including tutorials or guides. No new methodology nor theory is presented.

    - CookKF_2005_HSRes_A: *Objective. To provide an introduction to the use of CAT in the measurement of health outcomes, describe several IRT models that can be used as the basis of CAT, and discuss practical issues associated with the use of adaptive scaling in research settings.*
    - Siemons_2014_CERheu: *The aim is to familiarise physicians and researchers with the most important concepts of item response theory (IRT) and with its usefulness for improving test administration and data collection in health care.*
    - Hobart_2002_CONeu: *This review examines recent information in the area of quantifying patients' perspectives.*
    - Velozo_2012_APMR: *The purpose of this article is to present the clinician and researcher with a contemporary 8-stage framework for measurement scale development based on a mixed-methods qualitative and quantitative approach. Core concepts related to item response theory are presented.*

4. Applied: RMT/IRT models are applied (or mention that they have been applied in previous articles) to validate/co-calibrate/develop an instrument, or using the scores for further analyses.

    - Abe_2015_AJO: *In addition, the 2 items belonging to the subscale of ocular pain were also excluded, as they could not fit into the Rasch model.*
    - YanZi_2015_TTeaEdu_C: *A total of 450 teachers from 10 primary schools were surveyed. Teachers' responses to the scales were calibrated using Rasch analysis and then subjected to path analysis.*
    - PrietoL_2003_ActaPsySca: *A Rasch model analysis to test the cross-cultural validity of the EuroQoL-5D in the Schizophrenia Outpatient Health Outcomes Study.*
    - LundgrenNilsson_2011_JRehMed: *The current study has shown that the FIMTM Motor Scale, as applied to a stroke rehabilitation sample, satisfies Rasch model expectations and the unidimensionality assumptions, having accommodated local dependency issues, and by using the partial credit parameterization with re-scored categories.*
    - FredrikssonLarsson_2015_JNurMea: *The purpose of this study is to psychometrically evaluate the Multidimensional Fatigue Inventory (MFI-20). Methods: The MFI-20 was evaluated using Rasch analysis.*
    - Elhai_2011_JAD: *In the present paper, the authors tested whether PTSD and MDD are similar or unique constructs by examining their symptoms using Rasch modeling.*
    - Graticelli_2015_JAMAOph: *A joint model was used to investigate the association between change in NEI VFQ-25 Rasch-calibrated scores and change in RNFL thickness, adjusting for confounding socioeconomic and clinical variables.*
    - Cella_2014_QoLR: *Consensus was reached for anxiety on vignette ranking and severity cut scores with minimal discussion. For depression, discussion centered on whether the T score = 70 card should be "'moderate"' or "'severe;"' in the end it was retained in the "'moderate"' category.*

- CookKF_2015_QoLR_A: *Clinical vignettes were created at 0.5 standard deviations intervals (five points on T score metric). Because the score range varied by bank, the number of vignettes per domain also varied. For fatigue, nine T score locations were selected, ranging from 32.5 to 72.5. For sleep disturbance, there were 10 T score locations (range 32.0-84.0).*

5. Miscellaneous: RMT/IRT is clearly not the main focus (mentioned e.g. in the introduction or discussion sections of the article) or not applied.

   - Aguado_2014_GroDyn: *Moreover, the use of advanced psychometric models like those based on item response theory would allow the design of computerized adaptive tests able to optimize the administration of a wide-ranging measure.*
   - Ivanova_2013_Aphasiol: *An in-depth account of instrument development based on Rasch modeling is beyond the scope of this tutorial; for further information on the topic see Baylor et al. (2011), Bond and Fox, Embertson and Riese (2000), and Wolfe and Smith (2007a, 2007b).*
   - Zhao_2014_Entropy: *The family of multivariate binary distributions has been proven to be useful when we deal with discrete data in a variety of applications in statistical machine learning and artificial intelligence, such as the Boltzmann machine in neural networks and the Rasch model in human sciences.*
   - Chung_2015_QoLR: *The PROMIS-D-8 (8b short form) consists of eight items derived from an item bank scored using item response theory that measure cognitive and affective symptoms. [...] Future studies should investigate whether the findings of the current study are supported using other approaches, such as item response theory, in which measurement invariance is evaluated at the item level.*

Some articles were excluded during the Paradigm/Type allocation procedure because the construct assessed was out of social/health/educational sciences.
